# Zhang equivalency of inequation-to-inequation type for constraints of redundant manipulators

**DOI:** 10.1016/j.heliyon.2023.e23570

**Published:** 2023-12-12

**Authors:** Dongqing Wu, Yunong Zhang

**Affiliations:** a School of Computational Science, Zhongkai University of Agriculture and Engineering, Guangzhou 51220, Guangdong, China; b Research Institute of Sun Yat-sen University in Shenzhen, Sun Yat-sen University, Shenzhen 518057, Guangdong, China; c School of Computer Science and Engineering, Sun Yat-sen University, Guangzhou 510006, Guangdong, China

**Keywords:** Zhang equivalency, Time-dependent optimization motion planning, Inequation constraints, Quadratic programming, Redundant manipulator

## Abstract

In solving specific problems, physical laws and mathematical theorems directly express the connections between variables with equations/inequations. At times, it could be extremely hard or not viable to solve these equations/inequations directly. The PE (principle of equivalence) is a commonly applied pragmatic method across multiple fields. PE transforms the initial equations/inequations into simplified equivalent equations/inequations that are more manageable to solve, allowing researchers to achieve their objectives. The problem-solving process in many fields benefits from the use of PE. Recently, the ZE (Zhang equivalency) framework has surfaced as a promising approach for addressing time-dependent optimization problems. This ZEF (ZE framework) consolidates constraints at different tiers, demonstrating its capacity for the solving of time-dependent optimization problems. To broaden the application of ZEF in time-dependent optimization problems, specifically in the domain of motion planning for redundant manipulators, the authors systematically investigate the ZEF-I2I (ZEF of the inequation-to-inequation) type. The study concentrates on transforming constraints (i.e., joint constraints and obstacles avoidance depicted in different tiers) into consolidated constraints backed by rigorous mathematical derivations. The effectiveness and applicability of the ZEF-I2I are verified through two optimization motion planning schemes, which consolidate constraints in the velocity-tier and acceleration-tier. Schemes are required to accomplish the goal of repetitive motion planning within constraints. The firstly presented optimization motion planning schemes are then reformulated as two time-dependent quadratic programming problems. Simulative experiments conducted on the basis of a six-joint redundant manipulator confirm the outstanding effectiveness of the firstly presented ZEF-I2I in achieving the goal of motion planning within constraints.

## 1 Introduction

There are many applications of the PE (principle of equivalence) in different disciplines [1]. For instance, in physics, Albert Einstein firstly presented a PE on the basis of two equal masses: a noninertial frame is M&P (mathematically and physically) equivalent to an accelerated frame with a uniform gravitational field [2]. The importance of PE lies in its disregard for the constraint categories of physical systems and offering a consolidated standard to measure, compute, and control the physical quantities or geometric quantities of interest. This simplifies the complex properties of the model. In view of the universality of PE, scholars have initiated the development of approaches based on PE to address problems. The PE has been applied to obtain the solutions of a number of optimization problems as follows. Aiming to perform the on-orbital servicing tasks under strict environmental constraints, Hu et al. investigated a kinematic PE and applied it to the pose-configuration planning problem of a redundant manipulator (RM) [3]. In [4], a balancing technique based on Ma equivalence was firstly presented to obtain optimization solutions for RMs. Furthermore, researchers compared and utilized Ma equivalence to save kinetic energy or to achieve specific optimization objectives in robotics [5].

**Figure 1:**
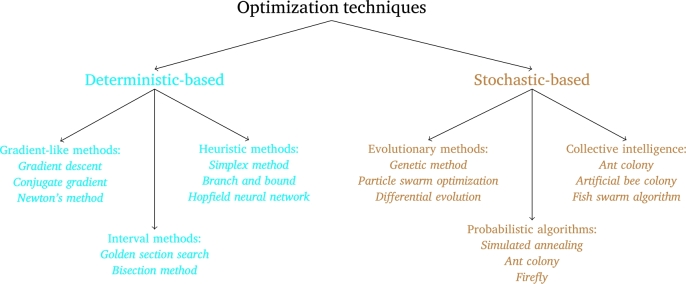
Taxonomy of optimization techniques.

In order to make the research more self-descriptive, a comprehensive review of optimization techniques is carried out due to the commonly found application of PE in optimization problems. Optimization is a primary process in different fields, including engineering and economics, that strives to identify the optimal solution from a range of viable alternatives [6], [7], [8], [9]. The main purpose of optimization is to pursue objective(s) within constraints. Optimization methods have undergone substantial advancements to tackle the difficulties presented by intricate and nonlinear problems. A taxonomy of optimization techniques is illustrated in Fig. 1. Optimization techniques can be classified into two major categories: deterministic-based techniques and stochastic-based techniques [6], [10]. In the category of deterministic-based, there exist approaches like gradient descent, conjugate gradient, and Newton's method, all of which belong to the family of gradient-based methods [11]. Furthermore, the deterministic techniques category encompasses interval methods such as golden section search and bisection [7], as well as heuristic methods, including the simplex method, branch and bound, and Hopfield NN (neural network) [8]. In the category of stochastic-based, there exist evolutionary methods such as genetic method, particle swarm optimization, and differential evolution [12]. Moreover, the stochastic-based optimization techniques category also comprises probabilistic algorithms like simulated annealing, ant colony algorithm, and firefly algorithm [9], as well as collective intelligence algorithms including ant colony, artificial bee colony, and fish swarm algorithm [13]. When selecting optimization techniques, it is crucial to consider various factors, including the attributes of the problem, the nature of the objective, and the constraints imposed by the problem. Deterministic-based techniques are typically well-suited for optimization problems in which gradient information is accessible, and they often exhibit faster convergence speeds. In contrast, stochastic-based techniques are better suited for optimization problems that are nonsmooth, multimodal, and highly nonlinear. These techniques possess global search capabilities and robustness, making them more effective in such scenarios. The incorporation of optimization techniques into diverse disciplines, including machine learning, uncertainty modeling, and robotics, has significantly broadened the range of applications and enhanced the capabilities of optimization methods.

RM refers to a robotic arm system that has an excess of DoF (degrees of freedom) beyond the minimum required to manipulate the end-effectors within the workspace [14], [15]. Due to these additional DoFs, some difficult tasks for RMs, e.g., joint physical limitation avoidance [16], OA (obstacles avoidance) [17], cyclic motion planning control [18], and fault tolerance [19], can be completed with the RMs. In recent years, the inverse-kinematics problem has been a hotspot in the optimization motion planning of RMs [20]. In industrial scenarios, the end-effector is required to track the predetermined path [21]. Therefore, the trajectories of joints need to be computed online [22]. The RMs are physically constrained by different limitations, e.g., position limitations, joint velocity limitations, and joint acceleration limitations [16]. As a result, ensuring all joints are within a reasonable scope during task execution is critical. If the motion of any joint surpasses the physical limitations, the manipulators will be damaged. Research on ensuring that joint motion remains within physical limitations has attracted significant interest in theoretical and practical fields [23]. Valtazanos et al. firstly presented an approach for the control of multi-robot subject to constraints physically [24]. In [25], Ruchanurucks presented a filtering method to optimize the trajectories of a humanoid robot with physical limitations. In addition, researchers have presented their intelligence systems for real-time solutions to the modeling planning problem of RMs with physical constraints [26], [27].

OA is an essential objective of task completion. Early research involved removing potential obstacles from the RMs' workspace [14]. Certainly, this approach restricts the performance of RMs in unpredictable environments. More innovative alternatives have concentrated on position-tier, velocity-tier, acceleration-tier, and jerk-tier for the manipulator in the constraints of multiple goals, including the criteria for OA [17], [28], [29], [15]. Among these alternative investigations, the constraints, e.g., physical limitations or OA, are abstracted as corresponding equations/inequations. The manipulator control scheme is usually formulated as a QP (quadratic programming) to determine the optimization objectives. For example, Zhang et al. investigated a neural-dynamic motion planning scheme and applied it to humanoid robot control [30]. Chen et al. provided a jerk-tier control scheme to compensate for the drift phenomenon of joint-angle for RM control with physical limitations [31]. In [32], Zhang et al. discussed the control optimization of RMs with joint physical constraints and firstly presented a novel QP scheme for complex path planning. Aiming better at noise-suppression, Liao and Xiang presented a discrete-time neural solution for QP subject to dynamic linear-equality constraints [33]. In the above works, the multiple objectives and constraints (i.e., physical limitations and OA) of the RM are discussed in different tiers and summarized into equations/inequations to obtain a feasible solution to the QP problem. Since they strongly rely on well-tuned parameters and lack a consolidated theoretical framework, these approaches may need more in-depth research and popularization of the results. RNNs (recurrent neural networks) are a research hotspot in many disciplines [34], [35]. In 2001, Zhang et al. conducted pioneering research and firstly presented a special category of RNN described as the ZNN (Zhang neural network) for online time-dependent equation solving. ZNN has been applied in a number of studies [36], [37], [38].

On the basis of ZNN, Zhang et al. newly established a theoretical framework of equivalency, i.e., ZEF (Zhang equivalency framework), especially for equality equivalency [5], [39]. A number of studies, e.g., [5], [20], [40], have employed the ZEF and obtained remarkable achievements. Most of the previous studies focus on investigating the ZEF of equation-to-equation (ZEF-E2E) type. In the ZEF-E2E, the constraints of the system are readily formulated as a time-dependent linear system, which is solved with the help of the Lagrange multiplier method [41]. Normally, the constraints in optimization problems, such as physical limitations and OA, are described as inequations. To exploit the method described in [41], one can transform the constraints in the form of inequations into constraints in the form of equations by introducing an unknown time-dependent adjustment function. The Lagrange multiplier method can be utilized to solve the equations derived from the constraints. The underlying assumption of this transformation is that the problem exhibits convexity within the feasible region, although this condition may not hold in certain scenarios [42], [43].

The ZEF of inequation-to-inequation (ZEF-I2I) type has emerged as a significant tool for tackling issues related to inequation-related problems. Offering a transformative approach allows for the conversion of one inequation into an equivalent form, which in turn facilitates the utilization of more effective problem-solving strategies. This paper comprehensively explores the framework of the inequation-to-inequation transformation using the ZEF-I2I approach, covering various aspects such as the current state, critical analysis, historical context, theoretical foundations, and model development. While ZEF-I2I has shown its effectiveness in numerous applications, it does have certain limitations. The effectiveness of ZEF-I2I is highly dependent on the assumptions. There may be instances where these assumptions are not valid, resulting in inaccuracies in the equivalency transformations. The ZEF-I2I may face difficulties when dealing with inequations that exhibit highly nonlinear behavior or complex constraints that necessitate the use of more advanced modeling techniques.

**Figure 2:**
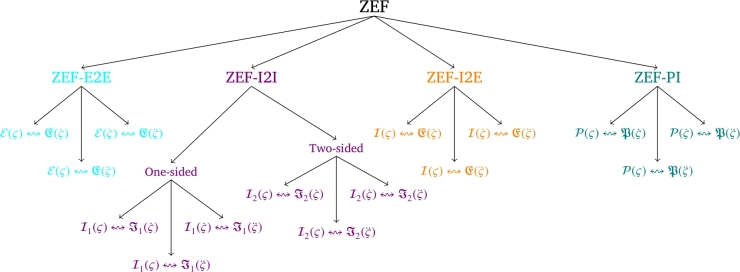
Taxonomy of ZEF (Note: *ς* denotes a time-dependent variable, $\overset{˙}{\varsigma }$ denotes first-order temporal derivative of *ς*, $\overset{¨}{\varsigma }$ denotes second-order temporal derivative of *ς*; symbol ↭ denotes that the equation/inequation on the left-hand part is M&P equivalent to the equation/inequation in the right-hand side; E(⋅) and E(⋅) represent different equations, I(⋅) and I(⋅) represent different inequations; $I_{1}(\cdot )$ and $I_{2}(\cdot )$ represent one-sided and two-sided inequations, respectively; P(⋅) and P(⋅) represent different PIs).

The origins of the ZEF-I2I can be traced back to the pioneering work of Zhang et al. [44]. Early investigations confirmed the mathematical approximations and practical equivalences of specific schemes developed utilizing the ZEF-I2I. In [40], [45], ZEF-I2I has been acknowledged as a valuable framework, and researchers have been exploring its applications in diverse domains. In [46], the theoretical foundation of ZEF-I2I comprises the mathematical formulation that enables the transformations between various inequations. ZEF-I2I is rooted in the fundamental principles of analyzing inequations, optimization theory, and mathematical modeling, forming the basis for its development and application. A profound comprehension of the mathematical principles and theory underpinning ZEF-I2I empowers researchers to devise inventive techniques for addressing problems related to inequations. Researchers have made considerable progress in creating models utilizing the ZEF-I2I framework. The development of models involves incorporating ZEF-I2I into diverse mathematical and computational frameworks. In general, the present state of ZEF-I2I highlights its crucial function in tackling challenges associated with inequations. Additionally, the ZEF-I2I possesses two notable advantages that the ZEF-E2E lacks. First, during optimization computation, ZEF-I2I accurately maintains the constraints with the same mathematical form, so it avoids the possible loss of accuracy caused by introducing the adjustment function. Second, the ZEF-I2I retains more comprehensive applications because the constrained feasible region of the constraints in the form of inequations is not required to be convex [47]. In order to augment comprehension of prior research on ZEF, the authors present a classification of it in Fig. 2. The first category (i.e., ZEF-E2E) was investigated in [48], [49]. In [50], ZEF-I2E (ZEF of inequation-to-equation) was explored and applied to address the issue of solving future linear inequations and equations across different tiers. In [51], a novel performance index for the acceleration-tier was introduced via ZEF-PI (ZEF of performance index) to facilitate configuration. In [52], ZEF-I2I was introduced as a means of addressing diverse problems related to time-dependent inequations. Furthermore, the ZEF-I2I approach was employed in the control of RMs [53]. However, subject to constraints posed by obstacles, existing schemes, such as those presented in [50], [51], [52], have shown instances where the joint acceleration surpasses its limitation. This is because the formulation of the scheme does not take into account the joint acceleration limitation. On this basis, gaps in the existing body of knowledge regarding the representation of constraints expressed as inequations in various tiers and their conversion into a consolidated tier are identified. Thus, a promising advancement could be achieved by consolidating the representation of inequation constraints, such as those related to OA, at the acceleration-tier. The present investigation is precisely motivated by this work.

Based on [5], a new model based on ZEF-I2I is firstly presented and applied to the motion planning of RMs from the position-tier to the velocity and acceleration-tiers. By bridging this gap, the field's comprehension is enhanced, and novel perspectives on time-dependent optimization methodologies are offered. First, a novel motion planning scheme for the RMs is firstly presented by integrating the novel model with the joint physical constraints. Next, the model is formulated as TDQP (time-dependent quadratic programming) constrained by equations/inequations. Thus, two schemes, i.e., the ZEF-I2I-VT (ZEF-I2I type in the velocity-tier) scheme and the ZEF-I2I-AT (ZEF-I2I type in the acceleration-tier) scheme, are put forward. The verification of the implemented schemes is achieved by meticulously scrutinizing their effectiveness through rigorous mathematical demonstrations and empirical evaluations. Experimental results based on a Kinova Jaco2 RM illustrate the effectiveness, superiority, and physical feasibility of the firstly presented schemes, i.e., ZEF-I2I-VT and ZEF-I2I-AT, in the control optimization of RMs. The primary innovation of this research lies in the systematic exploration and utilization of the ZEF-I2I in the realm of time-dependent optimization problems, with a specific emphasis on the transformation of OA constraints. The subsequent sections of this research are partitioned into six sections. Section 2 investigates and describes the ZEF-I2I. The general formulas of the ZEF-I2I in four typical cases are illustrated in Section 3. Section 4 discusses ZEF from the position-tier to the velocity-tier concerning different constraints. ZEF from the position-tier to the acceleration-tier is given in Section 5. In Section 6, the empirical results serve as additional evidence supporting the effectiveness and feasibility of the initially introduced schemes., i.e., ZEF-I2I-VT and ZEF-I2I-AT. Section 7 outlines the final remarks of this research. Below are the key contributions of this study:

1) To formulate the constraints of inequation type in widespread TDQP problems, the study investigates, derives, and provides the first-order and second-order general formulas of the ZEF-I2I type.

2) This study conducts a comprehensive survey, arrangement, and categorization of the ZEF. It also involves a meticulous analysis of the challenges intrinsic to this field.

3) This study presents an inequation equivalence strategy for OA, whereby position-tier constraints are transformed into equivalent velocity-tier and acceleration-tier constraints. This innovation provides a fresh perspective on addressing optimization problems with nonlinear constraints of a comparable type.

## 2 ZEF-E2E type

The ZEF-E2E type serves as the fundamental basis for the research framework in this study. To ensure the integrity and self-consistency of this study, the ZEF-E2E type is primarily concentrated on, and the corresponding formulas are presented in this section. Assume that one has the following time-dependent equation to pursue:

f(τ)=g(τ)∈R,

where f(τ) and g(τ) are time-dependent functions with time τ∈[0,+∞).

1) Zero-order general formula of ZEF-E2E type: Following the process of ZND (Zhang neural dynamics), one first defines the following error function:

(1) ϵ(τ)=f(τ)−g(τ)=0.

Equation (1) is also commonly known as the zero-order general formula of the ZEF-E2E type.

2) First-order general formula of ZEF-E2E type: Applying the Zhang design formula, i.e., $\overset{˙}{\epsilon }(\tau )=-\gamma \epsilon (\tau )$, one obtains the first-order general formula of ZEF-E2E type, which is formulated as

(2) $$\overset{˙}{\epsilon }(\tau )+\gamma \epsilon (\tau )=0,$$

in which $\overset{˙}{\epsilon }(\tau )$ represents the first-order temporal derivative of ϵ(τ) and *γ* is the design parameter. The closed-form solution to Eq. (2) is ϵ(τ)=ϵ(0)exp(−γt), where ϵ(0) represents the value of ϵ(τ) at τ=0. When both *τ* and *γ* are significantly greater than zero, one has ϵ(τ)=0, which is M&P equivalent to the zero-order general formula of ZEF-E2E type depicted in Eq. (1).

3) Second-order general formula of ZEF-E2E type: On the basis of Eq. (2), another error function is described, i.e., $\epsilon_{1}(\tau )=\overset{˙}{\epsilon }(\tau )+\gamma \epsilon (\tau )$. Substituting ϵ(τ) with $\epsilon_{1}(\tau )$ in Eq. (2), one obtains $\overset{¨}{\epsilon }(\tau )+\gamma \overset{˙}{\epsilon }(\tau )=-\gamma (\overset{˙}{\epsilon }(\tau )+\gamma \epsilon (\tau ))$ which is restated as

(3) $$\overset{¨}{\epsilon }(\tau )+2\gamma \overset{˙}{\epsilon }(\tau )+\gamma^{2}\epsilon (\tau )=0,$$

with $\overset{¨}{\epsilon }(\tau )$ denoting the second-order temporal derivative of ϵ(τ). One obtains the closed-form solution to Eq. (3) as

$$\epsilon (\tau )=(C_{1}+C_{2}t)\text{exp}(-\gamma t),$$

where $C_{1}=\epsilon (0)$ and $C_{2}=\overset{˙}{\epsilon }(0)+\gamma \epsilon (0)$. When both *τ* and *γ* are significantly greater than zero, ϵ(τ) in Eq. (3) approaches zero, i.e., ϵ(τ)=0, which is M&P equivalent to the zero-order general formula of ZEF-E2E type depicted in Eq. (1).

To gain a deeper understanding of the fundamental idea behind the ZEF-E2E, the general formulas of the ZEF-E2E type depicted in Eqs. (1)-(3) are collected as follows [39]:

(4a) ϵ(τ)=0,

(4b) $$\overset{˙}{\epsilon }(\tau )+\gamma \epsilon (\tau )=0,$$

(4c) $$\overset{¨}{\epsilon }(\tau )+2\gamma \overset{˙}{\epsilon }(\tau )+\gamma^{2}\epsilon (\tau )=0.$$

When both *τ* and *γ* are significantly greater than zero, the general formulas of the ZEF-E2E type depicted in Eqs. (4a)-(4c) are M&P equivalent to each other.

Remark 1 Note that the type of time-dependent variables in the above derivation process is uncertain, and the laws of scalar multiplication, addition, and subtraction are supported for time-dependent terms ϵ(τ), $\overset{˙}{\epsilon }(\tau )$, and $\overset{¨}{\epsilon }(\tau )$, whether the time-dependent terms are scalar-valued, vector-valued, or matrix-valued. Therefore, the general formulas of the ZEF-E2E type depicted in Eqs. (4a)-(4c) are evidently extended to various types of variables, e.g., scalar-valued, vector-valued, and matrix-valued, as in [5], [54]. According to the discussion in [39], the general formulas of the ZEF-E2E type depicted in Eqs. (4a)-(4c) can be generalized to higher-order temporal derivative forms, e.g., third-order and fourth-order.

## 3 ZEF-I2I type

In many control scenarios, the constraints of state-control variables are often defined as a number of time-dependent inequations [39]. This section discusses a more engineering-oriented ZEFGF (ZEF general formula) of inequation type. These time-dependent constraints are categorized into three situations presented and investigated in this section.

### 3.1 ZEFGFs of inequation type

This subsection provides the zero-order, first-order, and second-order ZEFGF for the ZEF of inequation type.

1) Zero-order ZEFGF of inequation type: Consider the following zero-order ZEFGF of inequation type:

(5) ϵ(τ)≤0,

where ϵ(τ)∈R represents a smooth function in $\left[0,\tau_{\text{f}}\right]$ ($\tau_{\text{f}}$ denotes the final time).

2) First-order ZEFGF of inequation type: Following the process of ZND, one describes the first-order ZEFGF of inequation type as presented below:

(6) $$\overset{˙}{\epsilon }(\tau )+\gamma \epsilon (\tau )\leq 0,$$

where *γ* is significantly greater than zero. Next, one proves that Eq. (6) is M&P equivalent to Eq. (5) with γ≫0, and subsequently introduces a proper time-dependent term, i.e., p(τ)≥0, which satisfies

(7) $$\overset{˙}{\epsilon }(\tau )+\gamma \epsilon (\tau )+p(\tau )=0.$$

Then, one obtains the closed-form solution to Eq. (7) as

$$\epsilon (\tau )=\text{exp}(-\gamma t)(\epsilon (0)-\int_{0}^{t}p(\tau )\text{exp}(\gamma \tau )\text{d}\tau ).$$

According to [52], with τ≥38/γ and γ=10000, ϵ(τ) can be viewed as 0 in Eq. (7). Therefore, the zero-order ZEFGF of inequation type depicted in Eq. (5) and the first-order ZEFGF of inequation type depicted in Eq. (6) are M&P equivalent.

3) Second-order ZEFGF of inequation type: Following a similar derivation process of the first-order ZEFGF of inequation type depicted in Eq. (6), one presents the second-order ZEFGF of inequation type as follows.

On the basis of Eq. (6), one defines another error function (i.e., $\epsilon_{1}(\tau )=\overset{˙}{\epsilon }(\tau )+\gamma \epsilon (\tau )$) and then expands ${\overset{˙}{\epsilon }}_{1}(\tau )+\gamma \epsilon_{1}(\tau )\leq 0$. Thus, one obtains the following second-order ZEFGF of inequation type:

(8) $$\overset{¨}{\epsilon }(\tau )+2\gamma \overset{˙}{\epsilon }(\tau )+\gamma^{2}\epsilon (\tau )\leq 0.$$

When both *τ* and *γ* are significantly greater than zero, the second-order ZEFGF of inequation type in Eq. (8) is M&P equivalent to the first-order ZEFGF of inequation type in Eq. (6), as well as the zero-order ZEFGF of inequation type in Eq. (5). To gain a deeper understanding of the fundamental idea behind the ZEF of inequation type, the above ZEFGFs of inequation type depicted in Eqs. (5), (6), and (8) are collected and reformulated as presented below:

(9a) ϵ(τ)≤0,

(9b) $$\overset{˙}{\epsilon }(\tau )\leq -\gamma \epsilon (\tau ),$$

(9c) $$\overset{¨}{\epsilon }(\tau )\leq -2\gamma \overset{˙}{\epsilon }(\tau )-\gamma^{2}\epsilon (\tau ).$$

When both *τ* and *γ* are significantly greater than zero, the ZEFGFs of inequation type depicted in Eqs. (9a)-(9c) are M&P equivalent to each other.

### 3.2 For bound constraints

First, one defines the following zero-order ZEF-I2I type for the bound constraint:

(10) $$\varsigma^{-}(\tau )\leq \varsigma (\tau )\leq \varsigma^{+}(\tau ),$$

in which $\varsigma^{-}(\tau )$, ς(τ), and $\varsigma^{+}(\tau )\in R$. To utilize the ZEFGFs of inequation type depicted in Eqs. (9a)-(9c), one reformulates the upper-bound part of Eq. (10) as

(11) $$\epsilon^{+}(\tau )=\varsigma (\tau )-\varsigma^{+}(\tau )\leq 0.$$

In a similar way, the lower-bound part of Eq. (10) is reformulated as

(12) $$\epsilon^{-}(\tau )=\varsigma^{-}(\tau )-\varsigma (\tau )\leq 0.$$

Combining Eqs. (11) and (12), one has the zero-order ZEF-I2I type as presented below:

$$\varsigma^{-}(\tau )-\varsigma (\tau )\leq 0,\varsigma (\tau )-\varsigma^{+}(\tau )\leq 0.$$

Following similar approaches and employing the ZEFGFs of inequation type depicted in Eqs. (9a)-(9c), one obtains the following first-order ZEF-I2I formulas for bound constraint in compact form:

(13) $${\overset{˙}{\varsigma }}^{-}(\tau )+\gamma (\varsigma^{-}(\tau )-\varsigma (\tau ))\leq \overset{˙}{\varsigma }(\tau )\leq {\overset{˙}{\varsigma }}^{+}(\tau )-\gamma (\varsigma (\tau )-\varsigma^{+}(\tau )),$$

and the second-order ZEF formulas for bound constraint in compact form:

(14) $${\overset{¨}{\varsigma }}^{-}(\tau )+2\gamma ({\overset{˙}{\varsigma }}^{-}(\tau )-\overset{˙}{\varsigma }(\tau ))+\gamma^{2}(\varsigma^{-}(\tau )-\varsigma (\tau ))\leq \overset{¨}{\varsigma }(\tau )\leq {\overset{¨}{\varsigma }}^{+}(\tau )-2\gamma (\overset{˙}{\varsigma }(\tau )-{\overset{˙}{\varsigma }}^{+}(\tau ))-\gamma^{2}(\varsigma (\tau )-\varsigma^{+}(\tau )).$$

To gain a deeper understanding of the fundamental idea behind the ZEF-I2I, the formulas depicted in Eqs. (10), (13), and (14) are collected as follows:

(15a) $$\varsigma^{-}(\tau )\leq \varsigma (\tau )\leq \varsigma^{+}(\tau ),$$

(15b) $${\overset{˙}{\varsigma }}^{-}(\tau )+\gamma (\varsigma^{-}(\tau )-\varsigma (\tau ))\leq \overset{˙}{\varsigma }(\tau )\leq {\overset{˙}{\varsigma }}^{+}(\tau )-\gamma (\varsigma (\tau )-\varsigma^{+}(\tau )),$$

(15c) $${\overset{¨}{\varsigma }}^{-}(\tau )+2\gamma ({\overset{˙}{\varsigma }}^{-}(\tau )-\overset{˙}{\varsigma }(\tau ))+\gamma^{2}(\varsigma^{-}(\tau )-\varsigma (\tau ))\leq \overset{¨}{\varsigma }(\tau )\leq {\overset{¨}{\varsigma }}^{+}(\tau )-2\gamma (\overset{˙}{\varsigma }(\tau )-{\overset{˙}{\varsigma }}^{+}(\tau ))-\gamma^{2}(\varsigma (\tau )-\varsigma^{+}(\tau )).$$

When both *τ* and *γ* are significantly greater than zero, the ZEF formulas for bound constraints depicted in Eqs. (15a)-(15c) are M&P equivalent to each other.

### 3.3 For less-than-or-equal-to-0 inequation constraint

First, one defines the following zero-order ZEF-I2I type for the less-than-or-equal-to-0 constraint:

ς(τ)≤0,

where ς(τ)∈R. Using the ZEFGF depicted in Eqs. (5), (6), and (8) of inequation type directly, one obtains the zero-order, first-order, and second-order ZEF-I2I type for less-than-or-equal-to-0 constraints below:

(16a) ς(τ)≤0,

(16b) $$\overset{˙}{\varsigma }(\tau )+\gamma \varsigma (\tau )\leq 0,$$

(16c) $$\overset{¨}{\varsigma }(\tau )+2\gamma \overset{˙}{\varsigma }(\tau )+\gamma^{2}\varsigma (\tau )\leq 0.$$

When both *τ* and *γ* are significantly greater than zero, the ZEF formulas for less-than-or-equal-to-0 constraints depicted in Eqs. (16a)-(16c) are M&P equivalent to each other.

### 3.4 For greater-than-or-equal-to-0 inequation constraint

First, one defines the following zero-order ZEF-I2I type for the greater-than-or-equal-to-0 constraint:

(17) ς(τ)≥0,

where ς(τ)∈R. To utilize the ZEFGF of inequation type depicted in Eqs. (9a)-(9c) directly, Eq. (17) is reformulated as −ς(τ)≤0. Thus, one obtains the zero-order, first-order, and second-order ZEF-I2I type for greater-than-or-equal-to-0 constraints as follows [39]:

(18a) −ς(τ)≤0,

(18b) $$-\overset{˙}{\varsigma }(\tau )-\gamma \varsigma (\tau )\leq 0,$$

(18c) $$-\overset{¨}{\varsigma }(\tau )-2\gamma \overset{˙}{\varsigma }(\tau )-\gamma^{2}\varsigma (\tau )\leq 0.$$

When both *τ* and *γ* are significantly greater than zero, the ZEF formulas for greater-than-or-equal-to-0 constraints depicted in Eqs. (18a)-(18c) are M&P equivalent to each other.

Remark 2 Consider that the type of time-dependent variables in the above derivation process is uncertain, and the laws of scalar multiplication, addition, and subtraction are supported for ς(τ), $\overset{˙}{\varsigma }(\tau )$, and $\overset{¨}{\varsigma }(\tau )$, whether ς(τ), $\overset{˙}{\varsigma }(\tau )$, and $\overset{¨}{\varsigma }(\tau )$ are scalar-valued, vector-valued, or matrix-valued. Therefore, the general formulas of the ZEF-I2I type described in Subsections 3.2 through 3.4 are clearly extended to various types of variables, e.g., scalar-valued, vector-valued, and matrix-valued, as in [5], [54]. Based on the discussion in [39], the general formulas of the ZEF-E2E type depicted in Eqs. (18a)-(18c) can be generalized to higher-order temporal derivative forms, e.g., third-order and fourth-order.

## 4 ZEF from position-tier to velocity-tier

The repetitive motion planning of the RMs should be subject to constraints [18]. At the end of each cycle, it is necessary for the end-effector to return to the initial position, the trajectory of the end-effector should maintain consistency with the desired path, the manipulators' physical limitations should be guaranteed, and the manipulator can avoid obstacles. Considering an *m*-link RM working in an *n*-dimensional (*n*-D) scene with *l* obstacles to avoid, the motion planning scheme is denoted as [18], [55]

(19) $$\text{objective:}\;{\left\|\phi (\tau_{\text{c}})-\phi (0)\right\|}_{2}^{2}\to 0,$$

(20) $$\text{s.}\;\text{t.}\;f(\phi (\tau ))\to r_{\text{d}}(\tau ),$$

(21) $$\max_{1\leq i\leq m}\{d_{\text{oc},i}(\tau )\}\leq d_{\text{outer}},$$

(22) $$\phi^{-}\leq \phi (\tau )\leq \phi^{+},$$

(23) $${\overset{¯}{\phi }}^{-}\leq \overset{˙}{\phi }(\tau )\leq {\overset{¯}{\phi }}^{+},$$

(24) $${\overset{´}{\phi }}^{-}\leq \overset{¨}{\phi }(\tau )\leq {\overset{´}{\phi }}^{+},$$

where each entry of ϕ(τ) denotes the angle of each joint at time *τ*, and $\tau_{\text{c}}$ denotes τ=kT in which *T* represents the duration of a single period of circular motion, and $k\in N^{+}$. In addition, $f(\cdot ):R^{m}\to R^{n}$ transforms the joint angle vector (or, more generally, the joint position vector) $\phi (\tau )\in R^{m}$ to the *n*-D coordinates of the end-effector of the manipulator, $r_{\text{d}}(\tau )$ denotes the desired path of the end-effector of the manipulator at time *τ*, $d_{\text{oc},i}(\tau )$ represents the minimum distance between the critical point in the *i*th link with obstacles at time *τ*, and $d_{\text{outer}}$ represents the allowed minimum safe distance between all links and obstacles. $\phi^{\pm }$, ${\overset{¯}{\phi }}^{\pm }$, and ${\overset{´}{\phi }}^{\pm }$ represent the minimum and maximum limitations of ϕ(τ), $\overset{˙}{\phi }(\tau )$, and $\overset{¨}{\phi }(\tau )$, respectively.

From the motion planning scheme depicted in Eqs. (19)-(24), one notices different expressions of constraints in different tiers (e.g., velocity-tier and acceleration-tier). For simplicity, objective depicted in Eq. (19) and constraints depicted in Eqs. (20)-(23) (note that the constraint depicted in Eq. (24) is considered in Section 5) of the position-tier are transformed into the velocity-tier constraints by applying ZEF-E2E formulas depicted in Eqs. (4a) and (4b) for equality constraints, as well as ZEF-I2I formulas depicted in Eqs. (15a) and (15b) for inequation constraints.

### 4.1 For end-effector task execution

The kinematics equation of an *m*-link RM is denoted as

$$f(\phi (\tau ))=r_{\text{a}}(\phi (\tau )),$$

where f(⋅) represents the kinematics transforming function and $r_{\text{a}}(\phi (\tau ))\in R^{n}$ represents the *n*-D coordinates of the actual end-effector. To ensure that the end-effector goes back to the initial position at time τ=kT and the trajectory of the end-effector aligns with the desired path, ${\left\|f(\phi (\tau ))-r_{\text{d}}(\tau )\right\|}_{2}$ is expected to zero out, with $r_{\text{d}}(\tau )\in R^{n}$ representing the desired path at time *τ*.

The constraint depicted in Eq. (20) is specified in the position-tier and is currently unsolvable. To transform the constraint depicted in Eq. (20) into an equation specified in the velocity-tier, one utilizes the ZEF-E2E formulas depicted in Eqs. (4a) and (4b). The first error function which quantifies the discrepancy between $r_{\text{d}}(\tau )$ and $r_{\text{a}}(\phi (\tau ))$ is given as

(25) $$e(\tau )=r_{\text{d}}(\tau )-r_{\text{a}}(\phi (\tau ))=0.$$

To obtain the equivalency of Eq. (25) in the velocity-tier, one exploits the ZEF-E2E formulas depicted in Eqs. (4a) and (4b) to Eq. (25) and gets

(26) $$J(\phi (\tau ))\overset{˙}{\phi }(\tau )={\overset{˙}{r}}_{\text{d}}(\tau )+\gamma (r_{\text{d}}(\tau )-r_{\text{a}}(\phi (\tau ))),$$

where $J(\phi (\tau ))=\partial f(\phi )/\partial \phi \in R^{n\times m}$ denotes the Jacobian of the kinematics mapping function. It is noticeable that Eq. (26) is M&P equivalent to $f(\phi (\tau ))\to r_{\text{d}}(\tau )$.

### 4.2 For joint physical limitations avoidance

In this subsection, one describes the mathematical expressions for joint physical limitations avoidance in terms of the ZEF-I2I from the position-tier to the velocity-tier. The mathematical expressions for joint physical limitations avoidance in the position-tier are

(27a) $$\phi^{-}(\tau )\leq \phi (\tau )\leq \phi^{+}(\tau ),$$

(27b) ϕ(τ)≤0,

(27c) ϕ(τ)≥0,

which represent the three cases of inequations, i.e., bound constraint, less-than-or-equal-to-0 constraint, and greater-than-or-equal-to-0 constraint in the position-tier, respectively. The mathematical expressions for joint physical limitations avoidance in the velocity-tier are

(28a) $${\overset{¯}{\phi }}^{-}(\tau )\leq \overset{˙}{\phi }(\tau )\leq {\overset{¯}{\phi }}^{+}(\tau ),$$

(28b) $$\overset{˙}{\phi }(\tau )\leq 0,$$

(28c) $$\overset{˙}{\phi }(\tau )\geq 0.$$

Equations (28a)-(28c) represent the three cases of inequations, i.e., bound constraint, less-than-or-equal-to-0 constraint, and greater-than-or-equal-to-0 constraint in the velocity-tier, respectively. According to the ZEF-I2I formulas depicted in Eqs. (15a) and (15b), the position constraints depicted in Eqs. (27a)-(27c) are M&P equivalent to

(29a) $$\gamma (\phi^{-}(\tau )-\phi (\tau ))+{\overset{˙}{\phi }}^{-}(\tau )\leq \overset{˙}{\phi }(\tau )\leq -\gamma (\phi (\tau )-\phi^{+}(\tau ))+{\overset{˙}{\phi }}^{+}(\tau ),$$

(29b) $$\overset{˙}{\phi }(\tau )+\gamma \phi (\tau )\leq 0,$$

(29c) $$-\overset{˙}{\phi }(\tau )-\gamma \phi (\tau )\leq 0.$$

Since the original constraints in the velocity-tier and transformed constraints in the velocity-tier must be satisfied at any given time, one combines Eqs. (28a)-(28c) with Eqs. (29a)-(29c) to obtain

$$\Phi_{\text{v}}^{-}(\tau )\leq \overset{˙}{\phi }(\tau )\leq \Phi_{\text{v}}^{+}(\tau ),\overset{˙}{\phi }(\tau )\leq \min\{0,-\gamma \phi (\tau )\},\overset{˙}{\phi }(\tau )\geq \max\{0,-\gamma \phi (\tau )\},$$

where $\Phi_{\text{v}}^{-}(\tau )=\max\{{\overset{¯}{\phi }}^{-}(\tau ),{\overset{˙}{\phi }}^{-}(\tau )+\gamma (\phi^{-}(\tau )-\phi (\tau ))\}$ and $\Phi_{\text{v}}^{+}(\tau )=\min\{{\overset{¯}{\phi }}^{+}(\tau ),{\overset{˙}{\phi }}^{+}(\tau )-\gamma (\phi (\tau )-\phi^{+}(\tau ))\}$.

### 4.3 For OA

For simplicity, each obstacle is abstracted as a point $o_{j}\in R^{n}$. Thus, all obstacles constitute an obstacle set $O={\left(o_{1}(\tau ),\cdots ,o_{l}(\tau )\right)}^{\text{T}}\in R^{l\times n}$ with *l* being the number of obstacles. If there are no occurrences of collisions between the RMs and the obstacles for every time *τ* during task duration *T*, the task of avoiding obstacles is successfully accomplished. Next, two steps are provided to formulate the OA into the constraint of the inequation type.

Step I: Find the critical point

**Figure 3:**
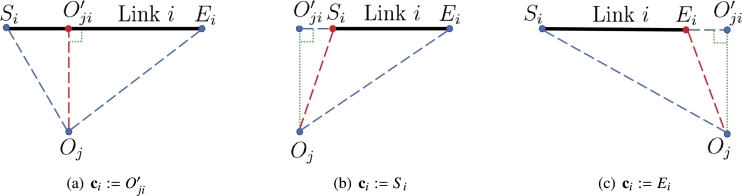
Situations of critical point ${\text{c}}_{i}\;(i=1,2,\cdots ,m)$ corresponding to three possible obstacle positions ${\text{o}}_{j}:=O_{j}\;(j=1,2,\cdots ,l)$ relative to link *i* (*i* = 1,2,⋯,*m*) of RM (red dashed line segment represents the minimum distance between obstacle *j* and link *i*).

The initial step in OA typically involves determining the critical point set $C=(c_{1}(\tau ),\cdots ,c_{m}(\tau ))$ on the manipulator with *m* links by finding the shortest distances between the obstacle set *O* and the *m* links at time *τ*. As illustrated in Figs. 3(a)-3(c), for the given link *i* and obstacle *j*, the position of the critical point on link *i* corresponding to obstacle *j*, which is denoted as $c_{i}^{j}(\phi (\tau ))\in R^{n}$, is thus determined by two determinants, i.e., $\Delta_{i1}$ and $\Delta_{i2}$ as follows [55]:

$$c_{i}^{j}(\phi (\tau )):=\left\{ \begin{array}{cc} & S_{i}(\phi (\tau )),\;\text{if}\;\Delta_{i1}\leq 0,\\ & E_{i}(\phi (\tau )),\;\text{if}\;\Delta_{i2}\leq 0,\\ & O_{ij}^{\prime }(\phi (\tau )),\text{if}\;\Delta_{i1}>0\;\text{and}\;\Delta_{i2}>0, \end{array}\right.$$

where $\Delta_{i1}=\varrho_{i1}^{2}+\varrho_{i3}^{2}-\varrho_{i2}^{2}$ and $\Delta_{i2}=\varrho_{i2}^{2}+\varrho_{i3}^{2}-\varrho_{i1}^{2}$, with $\varrho_{i1}$, $\varrho_{i2}$, and $\varrho_{i3}$ denoting the edge lengths of $△S_{i}O_{j}E_{i}$, i.e., $\varrho_{i1}=|S_{i}O_{j}|$, $\varrho_{i2}=|E_{i}O_{j}|$, and $\varrho_{i3}=|S_{i}E_{i}|$.

For link *i*, one finds the RM's critical point at time *τ* as follows:

ci(τ)=arg⁡minx1≤j≤l⁡‖x−oj(τ)‖2,

in which $x\in \left\{c_{i}^{1}(\phi (\tau )),c_{i}^{2}(\phi (\tau )),\cdots ,c_{i}^{l}(\phi (\tau ))\right\}$ denotes the critical point set on link *i* with i=1,2,⋯,m.

Step II: Constraint for inequation-based OA

One adopts the inequation-based collision-free constraint [55]:

(30) $$J_{\text{cf}}(\tau ,\phi (\tau ))\overset{˙}{\phi }(\tau )\leq 0,$$

where $J_{\text{cf}}(\tau ,\phi (\tau ))\in R^{\nu \sigma \times m}$ with ν=2 and 3, in the 2-D case and 3-D case, respectively, and *σ* being the pairs of impingement obstacles and critical points in the impingement link [14]. $J_{\text{cf}}(\tau ,\phi (\tau ))$ is further specified as

(31) $$J_{\text{cf}}(\tau ,\phi (\tau ))=-\text{sgn}(v_{\text{e}}(\tau ))⋄J_{\text{cp}}(\phi (\tau )),$$

in which sgn(⋅) denotes the vector-valued signum function, and vector $v_{\text{e}}(\tau )$ denotes the escape velocity from obstacle to the corresponding critical point (e.g., vector $\overset{\to }{O_{j}S_{i}}$ in Figs. 3(a)-3(c)):

$$v_{\text{e}}(\tau )=\frac{\text{d}}{\text{d}\tau }\left(o(\tau )-c(\phi (\tau ))\right).$$

The operator ⋄ in Eq. (31) is defined as

$$\alpha ⋄M=\left[\begin{array}{c} \alpha_{1}\mu_{1}\\ \alpha_{2}\mu_{2}\\ \vdots \\ \alpha_{w}\mu_{w} \end{array}\right]$$

where vector $\alpha ={\left[\alpha_{1},\alpha_{2},\cdots ,\alpha_{w}\right]}^{\text{T}}$, and $\mu_{i}$ represents the *i*th row vector of matrix *M*. Meanwhile, in Eq. (31), $J_{\text{cp}}(\phi (\tau ))\in R^{n\times m}$ denotes the Jacobian of critical point **c**.

Remark 3 The inequation-based collision-free constraint depicted in Eq. (30) requires the negative (i.e., escape direction) velocity of the critical point. This evidently leads to the increment of the distance ${\left\|c(\phi (\tau ))-o(\tau )\right\|}_{2}$. Therefore, the vulnerable link distances itself from the obstacle.

Noting the possible discontinuity in $\overset{˙}{\phi }(\tau )$ when the distance ${\left\|o(\tau )-c(\phi (\tau ))\right\|}_{2}$ equals $d_{\text{outer}}$, one introduces the smoothing process on the inequation-based constraint below [55]:

(32) $$J_{\text{cf}}(\tau ,\phi (\tau ))\overset{˙}{\phi }(\tau )\leq z_{\text{v}},$$

where the above smoothing process is imposed only when $d={\left\|o(\tau )-c(\phi (\tau ))\right\|}_{2}\leq d_{\text{outer}}$; $z_{\text{v}}=s(d)J^{+}$ with $J^{+}=\max\{J_{\text{cf}}(\tau ,\phi (\tau ))\overset{˙}{\phi }(\tau ){|}_{d=d_{\text{outer}}},0\}$. Considering the information provided in [55], the following smoothing function s(d) is precisely specified:

(33) $$s(d)=\left\{ \begin{array}{cc} 0, & \text{if}\;d\leq d_{\text{inner}},\\ \sin^{2}\left(\frac{0.5\pi (d-d_{\text{inner}})}{d_{\text{outer}}-d_{\text{inner}}}\right), & \text{if}\;d_{\text{inner}}<d<d_{\text{outer}},\\ 1, & \text{if}\;d\geq d_{\text{outer}}. \end{array}\right.$$

Remark 4 The smoothing function depicted in Eq. (33) is activated when link *i* enters the buffer zone $\left[d_{\text{inner}},d_{\text{outer}}\right]$ of the obstacle. That is, the even and gradual deceleration $J_{\text{cf}}(\tau ,\phi (\tau ))\overset{˙}{\phi }(\tau )$ is imposed through s(d). With the initial value of $J^{+}$, the left-hand side of Eq. (32) evidently vanishes. Therefore, the link is driven to avoid the obstacle when approaching the inner safety threshold $d_{\text{inner}}$.

### 4.4 QP formulation and solver

Combining the constraints discussed in Subsections 4.1 through 4.3, one considers the requirement that the end-effector must return to its starting position at time τ=kT, where *T* represents the duration of a single period and k=1,2,⋯. The objective is termed RMPC (repetitive motion planning and control) and is often solved with QP schemes [18], [55], which is formulated as

(34) ϕ(kT)−ϕ(0)=0.

Then, one refers to the ZEF-E2E formulas depicted in Eqs. (4a) and (4b) and obtains the equivalency of the objective depicted in Eq. (34) in the velocity-tier as

$$0.5{\left(\overset{˙}{\phi }(\tau )+p_{\text{v}}(\tau )\right)}^{\text{T}}\left(\overset{˙}{\phi }(\tau )+p_{\text{v}}(\tau )\right),$$

where $p_{\text{v}}(\tau )=\gamma_{1}(-\phi (\tau )+\phi (0))$.

Overall, the QP formulation of the scheme of the RM in the velocity-tier (i.e., ZEF-I2I-VT scheme) is M&P equivalent to the following TDQP problem [18]:

(35) $$\text{min.}\;0.5{\left(\overset{˙}{\phi }(\tau )+p_{\text{v}}(\tau )\right)}^{\text{T}}\left(\overset{˙}{\phi }(\tau )+p_{\text{v}}(\tau )\right),$$

(36) $$\text{s.}\;\text{t.}\;J(\phi (\tau ))\overset{˙}{\phi }(\tau )=b_{\text{v}}(\tau ),$$

(37) $$J_{\text{cf}}(\tau ,\phi (\tau ))\overset{˙}{\phi }(\tau )\leq z_{\text{v}},$$

(38) $${\overset{¯}{\Phi }}_{\text{v}}^{-}\leq \overset{˙}{\phi }(\tau )\leq {\overset{¯}{\Phi }}_{\text{v}}^{+},$$

where $b_{\text{v}}(\tau )={\overset{˙}{r}}_{\text{d}}(\tau )+\gamma (r_{\text{d}}-r_{\text{a}}(\phi (\tau )))$. PNN (projection neural network) provides an effective method for TDQP [18]. To facilitate the use of the PNN solver, one rewrites the above motion planning scheme in compact form as follows:

(39) $$\text{min.}\;0.5y_{\text{v}}^{\text{T}}Q_{\text{u}}y_{\text{v}}+p_{\text{v}}^{\text{T}}y_{\text{v}},$$

(40) $$\text{s.}\;\text{t.}\;A_{\text{v}}y_{\text{v}}=b_{\text{v}},$$

(41) $$C_{\text{v}}y_{\text{v}}\leq d_{\text{v}},$$

(42) $$y_{\text{v}}^{-}\leq y_{\text{v}}\leq y_{\text{v}}^{+},$$

where the time arguments *τ* in $y_{\text{v}}$, $p_{\text{v}}^{\text{T}}$, $A_{\text{v}}$, $b_{\text{v}}$, $C_{\text{v}}$, $y_{\text{v}}^{-}$, and $y_{\text{v}}^{+}$ are omitted for simplicity, and $y_{\text{v}}=\overset{˙}{\phi }(\tau )\in R^{m}$, $Q_{\text{u}}=I_{m}\in R^{m\times m}$, $A_{\text{v}}=J(\phi (\tau ))\in R^{n\times m}$, $b_{\text{v}}={\overset{˙}{r}}_{\text{d}}(\tau )+\gamma (r_{\text{d}}(\tau )-r_{\text{a}}(\phi (\tau )))\in R^{n}$, $C_{\text{v}}=J_{\text{cf}}(\tau ,\phi (\tau ))\in R^{\nu \sigma \times m}$, $d_{\text{v}}=z_{\text{v}}\in R^{\nu \sigma }$, $y_{\text{v}}^{-}=\Phi_{\text{v}}^{-}(\tau )$, and $y_{\text{v}}^{+}=\Phi_{\text{v}}^{+}(\tau )$.

Remark 5 To underscore the distinguishing features of PNNs, comprehensive elucidation is presented on the distinctions between PNNs and cutting-edge NNs, such as BCNN (block combined neural network) [56]. As indicated in Table 1, PNN and BCNN each possess unique architectures with variations in five aspects. Nevertheless, there are certain resemblances between these two categories of NNs. For instance, both PNN and BCNN demonstrate flexibility. PNN adapts the mapping by considering the data distribution to enhance discrimination in high-dimensional space. BCNN utilizes techniques such as parameter sharing or composite connections to adaptively acquire diverse feature representations. In terms of ensemble learning, BCNN integrates modules to enhance the comprehensiveness of feature depiction. Despite not directly combining modules, PNN achieves a similar result by increasing feature dimensions through high-dimensional projection.

**Table 1:** Comparison of PNN and BCNN.

Comparison	PNN	BCNN
Architecture	Layered architecture with explicit projection tiers	Combination of multiple NN modules
Objective	Feature extraction and pattern recognition	Enhanced model representation and learning capacity
Applications	Data visualization, dimensionality reduction, pattern recognition	Image processing, natural language processing, deep reinforcement learning
Adaptability	Automatic adjustment of mappings based on data distribution	Parameter sharing or combining of subnetworks
Combination learning	Expansion of feature dimensions through high-dimensional projection	Integration of modules for ensemble learning

Following the discussions in [57], [58], one adopts the PNN solver for the TDQP problem depicted in Eqs. (39)-(42) and presents two necessary lemmas to prove the effectiveness of the PNN solver in theory.

Lemma 1 *With the parameter*μ>0*employed to regulate the convergence speed of the PNN, and*κ≫0*denoting a sufficiently large positive real number, the PNN solver is presented in vector form as follows:*

(43) $${\overset{˙}{u}}_{\text{v}}=\mu \left(I+M_{\text{v}}^{\text{T}}\right)\left(P_{\Omega }({\overset{˜}{z}}_{\text{v}})-u_{\text{v}}\right),$$

*where the time arguments τ in*$u_{\text{v}}$*,*$M_{\text{v}}$*,*${\overset{˜}{z}}_{\text{v}}$*are omitted for simplicity;*${\overset{˜}{z}}_{\text{v}}=(I-M_{\text{v}})u_{\text{v}}-q_{\text{v}}$*, and*$u_{\text{v}}={\left[y_{\text{v}},g(\tau ),h(\tau )\right]}^{\text{T}}$*denotes the unknown vector;*$g(\tau )\in R^{m}$*and*$h(\tau )\in R^{\nu \sigma }$*denote the dual decision vectors determined by Eqs.*(40)*and*(41)*, respectively;*$q_{\text{v}}={\left[p_{\text{v}},-b_{\text{v}},d_{\text{v}}\right]}^{\text{T}}$*. Meanwhile,*$M_{\text{v}}$*,*$u_{\text{v}}^{-}$*, and*$u_{\text{v}}^{+}$*are given as*

$$M_{\text{v}}=\left[\begin{array}{ccc} Q_{\text{u}} & -A_{\text{v}}^{\text{T}} & C_{\text{v}}^{\text{T}}\\ A_{\text{v}} & O & O\\ -C_{\text{v}} & O & O \end{array}\right],u_{\text{v}}^{-}={\left[y_{\text{v}}^{-},-\kappa 1_{\text{g}},0\right]}^{\text{T}},\;\text{and}\;u_{\text{v}}^{+}={\left[y_{\text{v}}^{+},+\kappa 1_{\text{g}},+\kappa 1_{\text{h}}\right]}^{\text{T}}.$$

*At last, the projection operator*$P_{\Omega }(\cdot )$*is a linear piecewise function, and the kth entry of*$P_{\Omega }(\zeta )$*is given as*

(44) $${\left[P_{\Omega }(\zeta )\right]}_{k}=\left\{ \begin{array}{c} \zeta_{k}^{-},\;\text{if}\;\zeta_{k}\in (-\infty ,\zeta_{k}^{-}),\\ \zeta_{k},\;\;\text{if}\;\zeta_{k}\in [\zeta_{k}^{-},\zeta_{k}^{+}],\\ \zeta_{k}^{+},\;\text{if}\;\zeta_{k}\in (\zeta_{k}^{+},+\infty ), \end{array}\right.$$

*with*k=1,2,⋯,n+m+νσ*.*

Proof The proof can be extended by referring to the source cited as [36], [57]. □

Remark 6 The parameter *μ* influences the convergence speed of the PNN model. In the event that *μ* is excessively large, it might impede the algorithm from achieving convergence. In the event that *μ* is excessively small, it could lead to a slow convergence speed and the possibility of getting trapped in local minima. To tackle this issue, a trial-and-error approach described in [59] is employed, which utilizes a binary search-like technique to determine the optimum value for *μ*. For instance, preliminary estimations of the lower bound value $\mu_{\text{low}}^{\left(0\right)}$ and the upper bound value $\mu_{\text{up}}^{\left(0\right)}$ that enable the model to achieve convergence can be identified. Then, the new $\mu =(\mu_{\text{low}}^{\left(0\right)}+\mu_{\text{up}}^{\left(0\right)})/2$ is computed and applied to the model. If the model converges, *μ* is revised to $(\mu +\mu_{\text{up}}^{\left(0\right)})/2$, and further exploration is undertaken. In the event that the model fails to converge, *μ* is revised to $(\mu +\mu_{\text{low}}^{\left(0\right)})/2$. This iterative procedure persists until the maximum value of *μ* that enables the model to achieve convergence is determined.

Lemma 2 *Assume that the QP problem depicted in Eqs.* (39) *-* (42) *has the optimal solution* $y_{\text{v}}^{⁎}$ *. Initializing from any state* $u_{0}$ *, the unknown* **u** *of the PNN solver depicted in Eq.* (43) *converges toward its theoretical solution* $u^{⁎}(\tau )$ *, whose first m entries constitute the optimal solution* $y_{\text{v}}^{⁎}(\tau )$ *of QP problem depicted in Eqs.* (39) *-* (42) *at time τ.*

Proof The proof can be extended by referring to the source cited as [36], [57], [60]. □

Remark 7 Stability and convergence of PNN solver depicted in Eq. (43): When rank(J(ϕ(τ)))<m, PNN solver depicted in Eq. (43) exhibits stability according to Lyapunov theory and globally converges to a steady state. When rank(J(ϕ(τ)))=m, PNN solver depicted in Eq. (43) is globally exponentially stable and convergent to a distinct steady state. The aforementioned details can be extended based on Theorem 1 in [61].

Remark 8 Sensitivity analysis of PNN solver depicted in Eq. (43): Sensitivity analysis is a crucial aspect in validating certain significant values or parameters in NN-based research [62]. If the realization of the PNN solver depicted in Eq. (43) incorporates digital circuits, it would be valuable to investigate appropriate sampling intervals for the input instructions of the RM by performing sensitivity analysis at specific time intervals.

Remark 9 TC (time complexity) analysis of PNN solver depicted in Eq. (43): Suppose that the dimension of $u_{\text{v}}$ be $\overset{¯}{m}$ in PNN solver depicted in Eq. (43). The solver consists of two primary operations: The first primary operation involves the multiplication of the matrix $\left(I+M_{\text{v}}^{\text{T}}\right)$ with the vector $\left(P_{\Omega }({\overset{˜}{z}}_{\text{v}})-u_{\text{v}}\right)$, which has a TC of $O({\overset{¯}{m}}^{2})$. The second primary operation entails utilizing the Euler method to compute the $\overset{¯}{m}$ first-order linear ordinary differential equations. The TC for resolving each individual first-order linear differential equation is commonly $O(\overset{¯}{n})$, where $\overset{¯}{n}$ signifies the quantity of discrete time steps [63]. Hence, the TC for the second primary operation is $O(\overset{¯}{m}\overset{¯}{n})$. Taking into account the two primary operations, the overall TC of the algorithm is $O({\overset{¯}{m}}^{2})+O(\overset{¯}{m}\overset{¯}{n})=O(\overset{¯}{m}(\overset{¯}{m}+\overset{¯}{n}))$.

**Figure 4:**
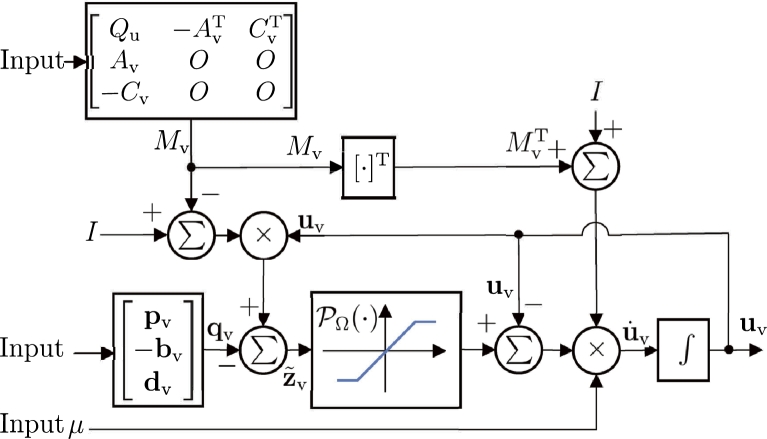
Block diagram of PNN solver depicted in Eq. (43).

The block diagram of the PNN solver depicted in Eq. (43) is presented in Fig. 4. The projection operator $P_{\Omega }:R^{n+m+\nu \sigma }\to \Omega$ serves as the operational amplifier, which can be implemented by using the so-called limiter [58]. As bound constraints in the position-tier and velocity-tier are consolidated in the velocity-tier, the size of the PNN solver depicted in Eq. (43) equals the dimension sum of constraints depicted in Eq. (40), constraints depicted in Eq. (41), and primal decision vector. The growth of the dimension of the problem is acceptable. Meanwhile, the PNN solver depicted in Eq. (43) reduces the TC of implementation compared to existing RNN-related models, owing to its purposely avoiding high-order computation, e.g., matrix inverse and matrix multiplication [64], [65].

## 5 ZEF from position-tier to acceleration-tier

In this section, the constraints in the position-tier and the velocity-tier are transformed into the constraints in the acceleration-tier by applying the ZEF-E2E and ZEF-I2I formulas presented in Sections 2 and 3, respectively.

### 5.1 For end-effector task execution

Applying the general formulas of ZEF-E2E type depicted in Eqs. (4a) and (4c) to $e(\tau )=r_{\text{d}}(\tau )-r_{\text{a}}(\phi (\tau ))$, one obtains the following equality constraint in second-order derivative form:

$$\overset{¨}{e}(\tau )+2\gamma \overset{˙}{e}(\tau )+\gamma^{2}e(\tau )=0.$$

One further gets

$$J(\phi (\tau ))\overset{¨}{\phi }(\tau )={\overset{¨}{r}}_{\text{d}}(\tau )-\overset{˙}{J}(\phi (\tau )){\overset{˙}{\phi }}^{2}(\tau )+2\gamma \left({\overset{˙}{r}}_{\text{d}}(\tau )-J(\phi (\tau ))\overset{˙}{\phi }(\tau )\right)-\gamma^{2}\left(r_{\text{d}}(\tau )-r_{\text{a}}\left(\phi (\tau )\right)\right),$$

where $\overset{˙}{J}$ represents the temporal derivative of *J*. Thus, one readily obtains the following equality constraint in the acceleration-tier:

(45) $$J(\phi (\tau ))\overset{¨}{\phi }(\tau )=b_{\text{a}}(\tau ),$$

in which $b_{\text{a}}(\tau )=-\overset{˙}{J}(\phi (\tau )){\overset{˙}{\phi }}^{2}(\tau )+{\overset{¨}{r}}_{\text{d}}(\tau )+2\gamma ({\overset{˙}{r}}_{\text{d}}(\tau )-J(\phi (\tau ))\overset{˙}{\phi }(\tau ))+\gamma^{2}(r_{\text{a}}(\phi (\tau ))-r_{\text{d}}(\tau ))$.

### 5.2 For joint physical limitations avoidance

This subsection describes the mathematical expressions for joint physical limitations avoidance in terms of the ZEF from the position-tier to the acceleration-tier.

According to the ZEF-I2I formulas depicted in Eqs. (15a)-(15c), one obtains the equivalency of the position constraint depicted in Eq. (22), the velocity constraint depicted in Eq. (23), and the acceleration constraint as follows:

$$\Phi_{\text{p2a}}^{-}(\tau )\leq \overset{¨}{\phi }(\tau )\leq \Phi_{\text{p2a}}^{+}(\tau ),\Phi_{\text{v2a}}^{-}(\tau )\leq \overset{¨}{\phi }(\tau )\leq \Phi_{\text{v2a}}^{+}(\tau ),\Phi_{\text{a2a}}^{-}(\tau )\leq \overset{¨}{\phi }(\tau )\leq \Phi_{\text{a2a}}^{+}(\tau ),$$

where $\Phi_{\text{p2a}}^{-}(\tau )={\overset{¨}{\phi }}^{-}(\tau )+2\gamma ({\overset{˙}{\phi }}^{-}(\tau )-\overset{˙}{\phi }(\tau ))+\gamma^{2}(\phi^{-}(\tau )-\phi (\tau ))$; $\Phi_{\text{p2a}}^{+}(\tau )={\overset{¨}{\phi }}^{+}(\tau )-2\gamma (\overset{˙}{\phi }(\tau )-{\overset{˙}{\phi }}^{+}(\tau ))-\gamma^{2}(\phi (\tau )-\phi^{+}(\tau ))$; $\Phi_{\text{v2a}}^{-}(\tau )={\overset{˙}{\overset{¯}{\phi }}}^{-}(\tau )+\gamma ({\overset{¯}{\phi }}^{-}(\tau )-\phi (\tau ))$; $\Phi_{\text{v2a}}^{+}(\tau )={\overset{˙}{\overset{¯}{\phi }}}^{+}(\tau )-\gamma (\phi (\tau )-{\overset{¯}{\phi }}^{-}(\tau ))$; $\Phi_{\text{a2a}}^{-}(\tau )={\overset{´}{\phi }}^{-}(\tau )$; and $\Phi_{\text{a2a}}^{+}(\tau )={\overset{´}{\phi }}^{+}(\tau )$. Therefore, the consolidated constraint in the acceleration-tier is synthesized as follows:

$$\Phi_{\text{a}}^{-}(\tau )\leq \overset{¨}{\phi }(\tau )\leq \Phi_{\text{a}}^{+}(\tau ),$$

with $\Phi_{\text{a}}^{-}(\tau )=\max\{\Phi_{\text{p2a}}^{-}(\tau ),\Phi_{\text{v2a}}^{-}(\tau ),\Phi_{\text{a2a}}^{-}(\tau )\}$ and $\Phi_{\text{a}}^{+}(\tau )=\text{min}\{\Phi_{\text{p2a}}^{+}(\tau ),\Phi_{\text{v2a}}^{+}(\tau ),\Phi_{\text{a2a}}^{+}(\tau )\}$.

### 5.3 For OA

The OA with the conventional ZEF method in the velocity-tier has been detailed in [55]. Referring to ZEF-I2I formulas depicted in Eqs. (15a) and (15c), studying this problem in the acceleration-tier is also feasible in terms of physics and mathematics. This subsection discusses this idea in detail.

Following the discussion in Subsection 4.3, the requirement for OA is formulated as an IC (inequation constraint) depicted in Eq. (30) in the velocity-tier. In this subsection, one applies the ZEF-I2I formulas depicted in Eqs. (15a) and (15c) to find the equivalency of Eq. (30) in the acceleration-tier. Although both sides of Eq. (30) may not be differentiable globally, it can be considered as differentiable locally, i.e., when $d\geq d_{\text{outer}}$. Therefore, one obtains the equivalency constraint of Eq. (30) in the acceleration-tier as

(46) $$J_{\text{cf}}(\tau ,\phi (\tau ))\overset{¨}{\phi }(\tau )\leq z_{\text{a}},$$

with $z_{\text{a}}=-{\overset{˙}{J}}_{\text{cf}}(\tau ,\phi (\tau ))\overset{˙}{\phi }(\tau )-\gamma (J_{\text{cf}}(\tau ,\phi (\tau ))\overset{¨}{\phi }(\tau )-z_{\text{v}})$. Inequation depicted in Eq. (46) is required to hold for each link at any time *τ*. Next, one provides a theorem to guarantee that the constraint depicted in Eq. (46) in the acceleration-tier, is M&P equivalent to achieving the goal of OA, i.e., ${\left\|c_{i}(\tau )-o_{j}(\tau )\right\|}_{2}^{2}\geq d_{j}^{2}$, for the *i*th link and any obstacle *j*.

Theorem 1 *Consider the OA requirement of an m-link RM. Assume that the theoretical solution*$\phi^{⁎}(\tau )$*for OA inequation exists. With differentiable terms*$J_{\text{cf}}(\tau ,\phi (\tau ))$*and*$z_{\text{a}}$*, sufficiently smooth critical point track*$c_{i}(\tau )$*, appropriately large convergence speed*$\gamma \in R^{+}$*, and sufficiently long time τ, IC depicted in Eq.*(46)*is M*&*P equivalent to*${\left\|c_{i}(\tau )-o_{j}(\tau )\right\|}_{2}^{2}\geq d_{j}^{2}$*.*

Proof Referring to the ZEF-I2I formulas depicted in Eqs. (16b) and (16c), IC depicted in Eq. (46) is clearly equivalent to IC depicted in Eq. (32). According to ZEF theory and Lemma 1 in [57], Eq. (32) is M&P equivalent to ${\left\|c_{i}(\tau )-o_{j}(\tau )\right\|}_{2}^{2}\geq d_{j}^{2}$. Therefore, one readily summarizes that IC depicted in Eq. (46) is M&P equivalent to ${\left\|c_{i}(\tau )-o_{j}(\tau )\right\|}_{2}^{2}\geq d_{j}^{2}$. The proof is thus completed.  □

### 5.4 QP formulation and PNN solver

Combining the constraints discussed in Subsections 5.1 through 5.3, one considers the aforementioned requirement that the end-effector must return to its starting position. The RMPC objective is solved with QP schemes [55]. To obtain the equivalency of the objective depicted in Eq. (34) in the acceleration-tier, one refers to the ZEF-E2E formulas depicted in Eqs. (4a) and (4c), and obtains the equivalency of the objective depicted in Eq. (34) in the acceleration-tier as

$$0.5{\left(\overset{¨}{\phi }(\tau )+p_{\text{a}}(\tau )\right)}^{\text{T}}\left(\overset{¨}{\phi }(\tau )+p_{\text{a}}(\tau )\right),$$

with $p_{\text{a}}(\tau )=-2\gamma \overset{˙}{\phi }(\tau )-\gamma^{2}(\phi (\tau )-\phi (0))$.

In summary, the QP formulation of the RM in the acceleration-tier (i.e., ZEF-I2I-AT scheme) is M&P equivalent to the TDQP problem below:

(47) $$\text{min.}\;0.5{\left(\overset{¨}{\phi }(\tau )+p_{\text{a}}(\tau )\right)}^{\text{T}}\left(\overset{¨}{\phi }(\tau )+p_{\text{a}}(\tau )\right),$$

(48) $$\text{s.}\;\text{t.}\;J(\phi (\tau ))\overset{¨}{\phi }(\tau )=b_{\text{a}}(\tau ),$$

(49) $$J_{\text{cf}}(\tau ,\phi (\tau ))\overset{¨}{\phi }(\tau )\leq z_{\text{a}},$$

(50) $${\overset{¯}{\Phi }}_{\text{a}}^{-}\leq \overset{¨}{\phi }(\tau )\leq {\overset{¯}{\Phi }}_{\text{a}}^{+},$$

where $b_{\text{a}}(\tau )$ is the same as that in Eq. (45). The QP problem depicted in Eqs. (47)-(50) is rewritten in compact form as follows:

(51) $$\text{min.}\;0.5y_{\text{a}}^{\text{T}}Q_{\text{u}}y_{\text{a}}+p_{\text{a}}^{\text{T}}y_{\text{a}},$$

(52) $$\text{s.}\;\text{t.}\;A_{\text{a}}y_{\text{a}}=b_{\text{a}},$$

(53) $$C_{\text{a}}y_{\text{a}}\leq d_{\text{a}},$$

(54) $$y_{\text{a}}^{-}\leq y_{\text{a}}\leq y_{\text{a}}^{+},$$

where the time arguments *τ* in $y_{\text{a}}$, $p_{\text{a}}$, $A_{\text{a}}$, $b_{\text{a}}$, $C_{\text{a}}$, $y_{\text{a}}^{-}$, and $y_{\text{a}}^{-}$ are omitted for simplicity, and $y_{\text{a}}=\overset{˙}{\phi }(\tau )$, $Q_{\text{u}}=I_{m}$, $A_{\text{a}}=J(\phi (\tau ))$, $C_{\text{a}}=J_{\text{cf}}(\tau ,\phi (\tau ))$, $d_{\text{a}}=z_{\text{a}}$, $y_{\text{a}}^{-}=\Phi_{\text{a}}^{-}$, and $y_{\text{a}}^{+}=\Phi_{\text{a}}^{+}$. Following the discussions in Subsection 4.5 and [57], [58], one presents the following PNN solver to solve the TDQP problem depicted in Eqs. (51)-(54):

(55) $${\overset{˙}{u}}_{\text{a}}=\mu (I+M_{\text{a}}^{\text{T}})(P_{\Omega }({\overset{˜}{z}}_{\text{a}})-u_{\text{a}}),$$

where the time arguments *τ* in ${\overset{˙}{u}}_{\text{a}}$, $M_{\text{a}}^{\text{T}}$, and $z_{\text{a}}$ are omitted for simplicity, and $z_{\text{a}}=(I-M_{\text{a}})u_{\text{a}}-q_{\text{a}}$ with

$$u_{\text{a}}=\left[\begin{array}{c} y_{\text{a}}\\ g(\tau )\\ h(\tau ) \end{array}\right],\;q_{\text{a}}=\left[\begin{array}{c} p_{\text{a}}\\ -b_{\text{a}}\\ d_{\text{a}} \end{array}\right],\;M_{\text{a}}=\left[\begin{array}{ccc} Q_{\text{u}} & -A_{\text{a}}^{\text{T}} & C_{\text{a}}^{\text{T}}\\ A_{\text{a}} & O & O\\ -C_{\text{a}} & O & O \end{array}\right],u_{\text{a}}^{-}=\left[\begin{array}{c} y_{\text{a}}^{-}\\ -\kappa 1_{\text{g}}\\ 0 \end{array}\right],\;\text{and}\;u_{\text{a}}^{+}=\left[\begin{array}{c} y_{\text{a}}^{+}\\ +\kappa 1_{\text{g}}\\ +\kappa 1_{\text{h}} \end{array}\right].$$

Evidently, the projection operator $P_{\Omega }(\cdot )$ denotes a linear piecewise function illustrated in Eq. (44).

## 6 Simulative experiments

In this section, simulative experiments are conducted using both the firstly presented schemes and the conventional method to validate their efficacy. The experimental results obtained by the two schemes are subsequently investigated and compared. The convergence speed *γ* is set to 5 in all experiments. Meanwhile, the period *T* of repetitive motion is fixed as 30 s. The computational platform and programming language employed is MATLAB 2014b. The experimental platform is a Kinova Jaco2 RM with six DoFs operating in 3D space. D-H (Denavit-Hartenberg) parameters provide a straightforward method for modeling the links and joints of RM [66]. Table 2 presents the D-H parameters of the Kinova Jaco2 RM [67]. The initial position $\phi (0)=1/60{\left[4\pi ,20\pi ,-5\pi ,15\pi ,10\pi ,5\pi \right]}^{\text{T}}$ rad. In addition, one gives the related parameters $\xi =10^{4}$ and $\varsigma =10^{10}$. The task of tracking is to draw a nephroid path, which is depicted as

(56) $$r_{\text{d}}(\tau )=\left[\begin{array}{c} x\\ y\\ z \end{array}\right]=\left[\begin{array}{c} 5\left(3\cos(\tau )-\cos(3\tau )\right)\\ 5\left(3\sin(\tau )-\sin(3\tau )\right)\\ 0 \end{array}\right].$$

**Table 2:** D-H Parameters of Kinova Jaco2 RM.

Joint	*ϕ*_*i*_ (rad)	*d*_*i*_ (mm)	*a*_*i*_ (mm)	*α*_*i*_ (rad)
1	*ϕ*_1_	275.5	0	*π*/2
2	*ϕ*_2_	0	410.0	*π*
3	*ϕ*_3_	−9.8	0	*π*/2
4	*ϕ*_4_	−250.1	0	*π*/3
5	*ϕ*_5_	−85.6	0	*π*/3
6	*ϕ*_6_	−157.8	0	*π*

Consider a fixed-position obstacle $o(\tau )={\left[-0.300,-0.050,0.350\right]}^{\text{T}}$ m. The safe inner distances for each link are $d_{\text{inner}}^{i}=0.05$ m with i=1,2,⋯,6. The safe outer distances for each link are $d_{\text{outer}}^{i}=0.15$ m with i=1,2,⋯,6.

### 6.1 From position-tier to velocity-tier

**Figure 5:**
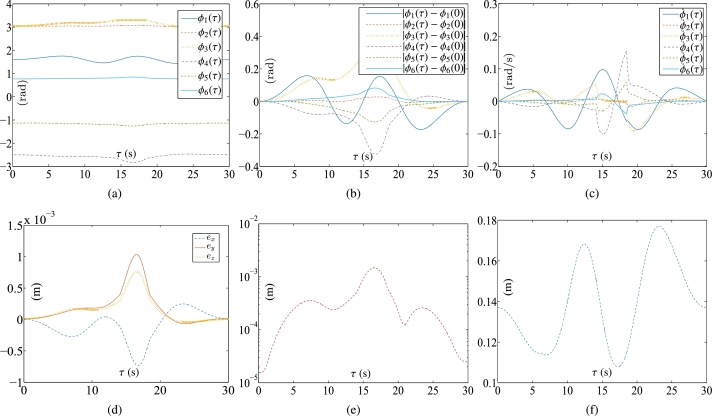
Synthesized results of Kinova Jaco2 RM obtained by PNN solver depicted in Eq. (43) with constraints depicted in Eqs. (40)-(42). (a) Trajectories of joint angle *ϕ*_*i*_(*τ*). (b) Trajectories of |*ϕ*_*i*_(*τ*)−*ϕ*_*i*_(0)|. (c) Trajectories of joint angle velocity ${\overset{˙}{\phi }}_{i}(\tau )$. (d) Trajectories of tracking errors in each axis. (e) Trajectory of ${\left\|r_{\text{d}}-r_{\text{a}}\right\|}_{2}$. (f) Trajectory of minimum link-obstacle distance.

**Figure 6:**
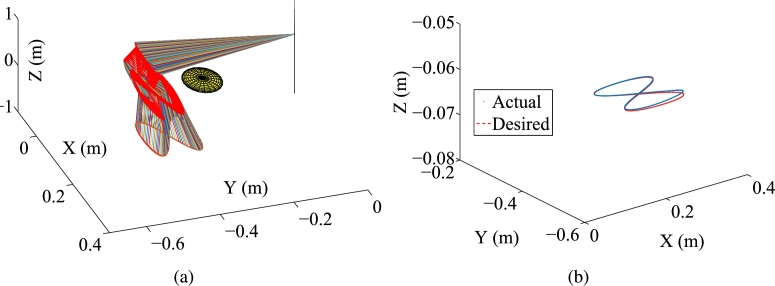
Motion trajectories of Kinova Jaco2 RM obtained by PNN solver depicted in Eq. (43) with constraints depicted in Eqs. (40) and (42). (a) Trajectory of manipulator. (b) Actual trajectory of end-effector and desired path.

**Figure 7:**
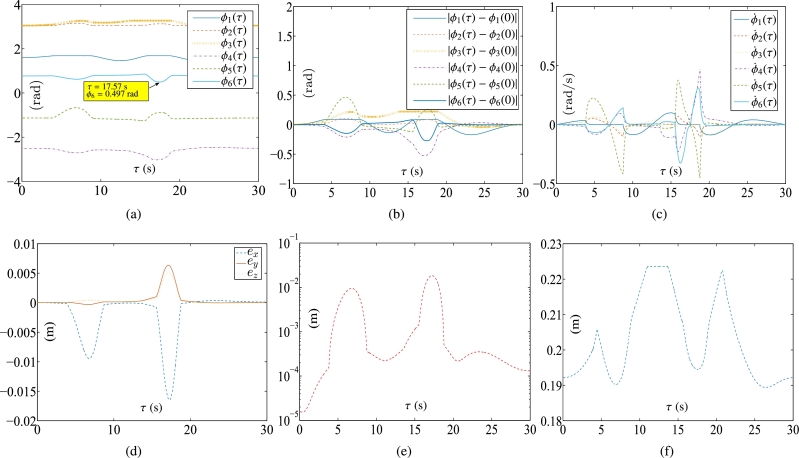
Synthesized results of Kinova Jaco2 RM obtained by PNN solver depicted in Eq. (43) with constraints depicted in Eqs. (40), (41), and (57). (a) Trajectories of joint angle *ϕ*_*i*_(*τ*). (b) Trajectories of |*ϕ*_*i*_(*τ*)−*ϕ*_*i*_(0)|. (c) Trajectories of joint angle velocity ${\overset{˙}{\phi }}_{i}(\tau )$. (d) Trajectories of tracking errors in X, Y, and Z axes. (e) Trajectory of ${\left\|r_{\text{d}}-r_{\text{a}}\right\|}_{2}$. (f) Trajectory of minimum link-obstacle distance.

**Figure 8:**
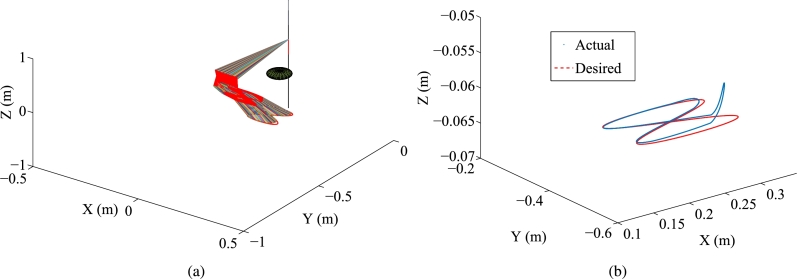
Motion trajectories of Kinova Jaco2 RM obtained by PNN solver depicted in Eq. (43) with constraints depicted in Eqs. (40), (41), and (57). (a) Trajectory of manipulator. (b) Actual trajectory of end-effector and desired path.

Some synthesized results from the Kinova Jaco2 RM using the PNN solver depicted in Eq. (43) to solve TDQP problem depicted in Eqs. (35)-(38) are displayed in Figure 5, Figure 6 by setting the sampling gap g=10 ms and using constraints depicted in Eqs. (39)-(42). The RM's physical limitations in this experiment are illustrated in Table 3. The plots in Figs. 5(a)-5(c) depict the trajectories of joint angles, joint angles' variations, and joint velocities, respectively. The physical constraints listed in Table 3 are affirmed by the observations from Figs. 5(a) and 5(c). The objective of cycle motion planning is achieved, as indicated in Fig. 5(b). Figs. 5(d) and 5(e) provide visual representations of the orders of maximal errors. The results of OA are satisfied, as shown in Fig. 5(f). Figs. 6(a) and 6(b) showcase the motion trajectories of the end-effector. In the previous works [57], [68], the conventional equivalency of bound constraints Eqs. (22) and (23) is presented as the following bound constraint:

(57) $${\overset{˜}{\Phi }}_{\text{v}}^{-}\leq y_{\text{v}}(\tau )\leq {\overset{˜}{\Phi }}_{\text{v}}^{+},$$

where ${\overset{˜}{\Phi }}_{\text{v}}^{-}=\max\{\lambda_{1}(\lambda_{2}\phi^{-}-\phi (\tau )),\lambda_{3}({\overset{˙}{\phi }}^{-}-\overset{˙}{\phi }(\tau ))\}$ and ${\overset{˜}{\Phi }}_{\text{v}}^{+}=\min\{\lambda_{1}(\lambda_{2}\phi^{+}-\phi (\tau )),\lambda_{3}({\overset{˙}{\phi }}^{+}-\overset{˙}{\phi }(\tau ))\}$ with $\lambda_{1}$, $\lambda_{2}$, $\lambda_{3}\in R^{+}$ serving as the adjustment parameters. It is noted that the adjustment parameters $\lambda_{1}$, $\lambda_{2}$, and $\lambda_{3}$ need to be set appropriately. Therefore, the conventional equivalency of bound constraints is not sufficiently effective. For comparison, one carries out the experiment for the conventional equivalency of bound constraints. The adjustment parameters are specified as $\lambda_{1}=5$, $\lambda_{2}=0.99$, and $\lambda_{3}=5$. The experimental results synthesized by the PNN solver depicted in Eq. (43) with bound constraint depicted in Eq. (57) are illustrated in Figs. 7(a)-7(f) and Figs. 8(a)-8(b). In Fig. 7(a), one joint angle, i.e., $\phi_{6}(\tau )$, is not bounded by the physical constraints prescribed in Table 3. Therefore, it is apparent that the conventional equivalency of bound constraints in the velocity-tier is not adequately effective.

**Table 3:** Physical limitations of RM in Subsection 6.1.

Joint	*ϕ*^−^ (rad)	$\phi^{+}$ (rad)	${\overset{¯}{\phi }}^{-}$ (rad/s)	${\overset{¯}{\phi }}^{+}$ (rad/s)	${\overset{´}{\phi }}^{-}$ (rad/s^2^)	${\overset{´}{\phi }}^{+}$ (rad/s^2^)
1	0.25*π*	1.7	−1.0	1.0	−0.5	0.5
2	0.5*π*	1.5*π*	−1.0	1.0	−0.5	0.5
3	0	3.3	−1.0	1.0	−0.5	0.5
4	−*π*	0	−1.0	1.0	−0.5	0.5
5	−0.5*π*	0.5*π*	−1.0	1.0	−0.5	0.5
6	0.5	0.5*π*	−1.0	1.0	−0.5	0.5

### 6.2 From position-tier to acceleration-tier

**Figure 9:**
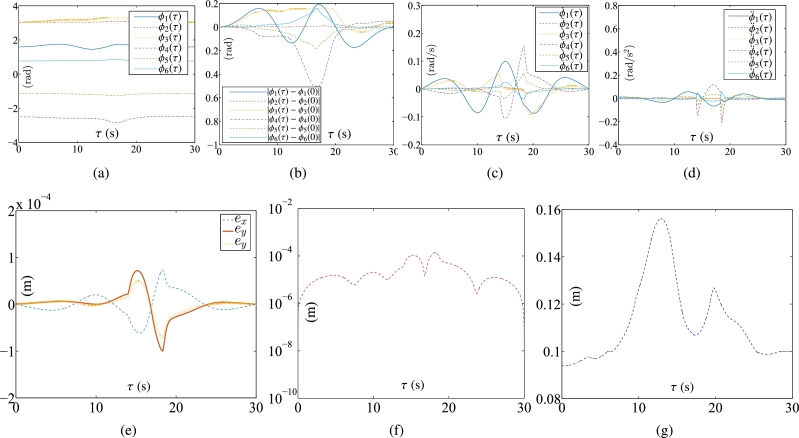
Synthesized results of Kinova Jaco2 RM obtained by PNN solver depicted in Eq. (55) with constraints depicted in Eqs. (52)-(54). (a) Trajectories of joint angle *ϕ*_*i*_(*τ*). (b) Trajectories of |*ϕ*_*i*_(*τ*)−*ϕ*_*i*_(0)|. (c) Trajectories of joint angle velocity ${\overset{˙}{\phi }}_{i}(\tau )$. (d) Trajectories of joint angle acceleration ${\overset{¨}{\phi }}_{i}(\tau )$. (e) Trajectories of tracking errors in X, Y, and Z axes. (f) Trajectory of ${\left\|r_{\text{d}}-r_{\text{a}}\right\|}_{2}$. (g) Trajectory of minimum link-obstacle distance.

**Figure 10:**
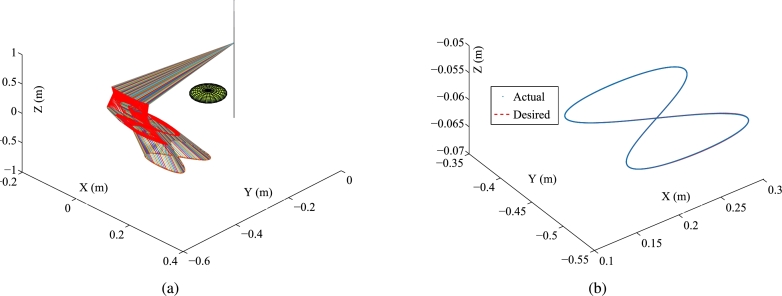
Motion trajectories of Kinova Jaco2 RM obtained by PNN solver depicted in Eq. (55) with constraints depicted in Eqs. (52)-(54). (a) Trajectory of manipulator. (b) Actual trajectory of end-effector and desired path.

Some synthesized results of the Kinova Jaco2 RM obtained by PNN solver depicted in Eq. (55) to solve the TDQP problem depicted in Eqs. (47)-(50) are displayed in Figure 9, Figure 10, with setting sampling gap g=10 ms. In particular, Fig. 10(a) illustrates the trajectory of the manipulator. Fig. 10(b) displays both the actual trajectory of the end-effector and the desired path. To compare the conventional method with the firstly presented ZEF-I2I-AT scheme, one prescribes the RM's physical limitations in this experiment in Table 4.

**Table 4:** Physical limitations of RM in Subsection 6.2.

Joint	*ϕ*^−^ (rad)	*ϕ*^+^ (rad)	${\overset{¯}{\phi }}^{-}$ (rad/s)	${\overset{¯}{\phi }}^{+}$ (rad/s)	${\overset{´}{\phi }}^{-}$ (rad/s^2^)	${\overset{´}{\phi }}^{+}$ (rad/s^2^)
1	0.25*π*	1.25*π*	−1.0	1.0	−0.5	0.5
2	0.5*π*	1.5*π*	−1.0	1.0	−0.5	0.5
3	0	3.2	−1.0	1.0	−0.5	0.5
4	−3.0	0	−1.0	1.0	−0.5	0.5
5	−0.5*π*	0.5*π*	−1.0	1.0	−0.5	0.5
6	−0.5*π*	1.35	−1.0	1.0	−0.5	0.5

**Figure 11:**
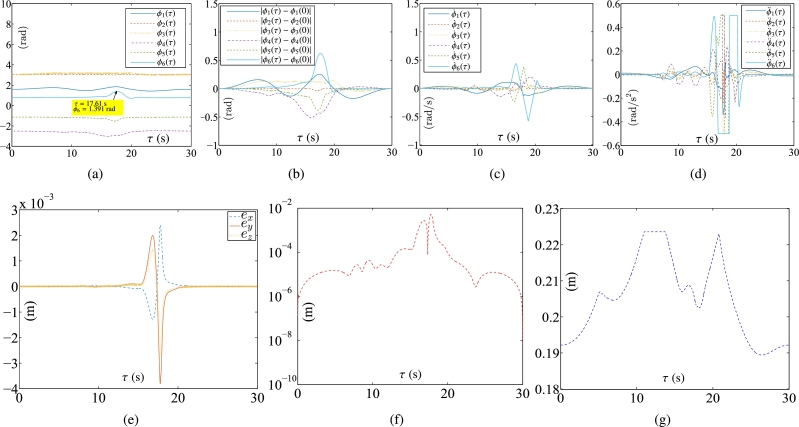
Synthesized results of Kinova Jaco2 RM obtained by PNN solver depicted in Eq. (55) with constraints depicted in Eqs. (52), (53), and (58). (a) Trajectories of joint angle *ϕ*_*i*_(*τ*). (b) Trajectories of |*ϕ*_*i*_(*τ*)−*ϕ*_*i*_(0)|. (c) Trajectories of joint angle velocity ${\overset{˙}{\phi }}_{i}(\tau )$. (d) Trajectories of joint angle acceleration ${\overset{¨}{\phi }}_{i}(\tau )$. (e) Trajectories of tracking errors in X, Y, and Z axes. (f) Trajectory of ${\left\|r_{\text{d}}-r_{\text{a}}\right\|}_{2}$. (g) Trajectory of minimum link-obstacle distance.

**Figure 12:**
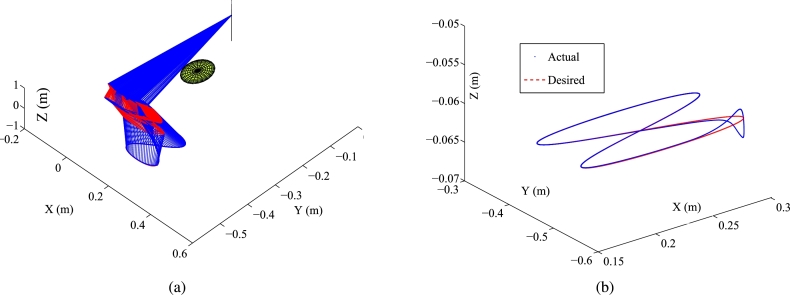
Motion trajectories of Kinova Jaco2 RM obtained by PNN solver depicted in Eq. (55) with constraints depicted in Eqs. (52), (53), and (58). (a) Trajectory of manipulator. (b) Actual trajectory of end-effector and desired path.

The plots in Figs. 9(a)-9(d) depict the trajectories of joint angles, joint angles' variations, joint velocities, and joint accelerations, respectively. The physical constraints listed in Table 2 are affirmed by the observations from Figs. 9(a), 9(c), and 9(d). The objective of cycle motion planning is achieved, as shown in Fig. 9(b). The maximal error orders and trajectories of ${\left\|r_{\text{d}}-r_{\text{a}}\right\|}_{2}$ are illustrated in Figs. 9(e) and 9(f), respectively. The results of OA are satisfied in Fig. 9(g). Additionally, in the previous works [68], [57], the conventional equivalency of bound constraints depicted in Eqs. (22)-(24) is presented as the following bound constraint:

(58) $${\overset{˜}{\Phi }}_{\text{a}}^{-}\leq y_{\text{a}}(\tau )\leq {\overset{˜}{\Phi }}_{\text{a}}^{+},$$

where ${\overset{˜}{\Phi }}_{\text{a}}^{-}=\max\{{\overset{˜}{\Phi }}_{\text{v}}^{-},\;{\overset{¨}{\phi }}^{-}\}$ and ${\overset{˜}{\Phi }}_{\text{a}}^{+}=\min\{{\overset{˜}{\Phi }}_{\text{v}}^{+},\;{\overset{¨}{\phi }}^{+}\}$ with ${\overset{˜}{\Phi }}_{\text{v}}^{-}$ and ${\overset{˜}{\Phi }}_{\text{v}}^{+}$ being defined in Eq. (57). It is noted that the bound constraint depicted in Eq. (58) requires the adjustment parameters to be set appropriately. For comparison, one carries out an experiment for the conventional equivalency of bound constraint depicted in Eq. (58). The adjustment parameters are set as $\lambda_{1}=5$, $\lambda_{2}=0.99$, and $\lambda_{3}=5$, respectively. Some synthesized results of the Kinova Jaco2 RM using the PNN solver depicted in Eq. (55) with bound constraint depicted in Eq. (58) are presented in Figs. 11(a)-11(g). In Fig. 11(a), one joint angle, i.e., $\phi_{6}(\tau )$, is not bounded by the physical constraints detailed in Table 4. Figs. 12(a) and 12(b) showcase the manipulator's trajectory, along with the desired path and the actual trajectory of the end-effector obtained by using the PNN solver depicted in Eq. (55). Table 5 displays the MTE (mean tracking error), represented as ${\left\|r_{\text{d}}-r_{\text{a}}\right\|}_{2}$, for ten randomly selected sets of initial angles q(0). Every row includes the six initial angles $\phi_{1}(0)$ to $\phi_{6}(0)$ and the associated MTE ${\left\|r_{\text{d}}-r_{\text{a}}\right\|}_{2}$ in meters. Table 6 describes the RMPC errors (i.e., $\delta_{i}={\left\|\phi (kT)-\phi (0)\right\|}_{2}$), obtained by PNN solver depicted in Eq. (55) for randomly selected initial angles ***ϕ*** at τ=0 s.

**Table 5:** MTE ${\left\|r_{\text{d}}-r_{\text{a}}\right\|}_{2}$ computed by PNN solver depicted in Eq. (55) with random initial angle ***ϕ*** at *τ* = 0 s.

No.	*ϕ*_1_(0) (rad)	*ϕ*_2_(0) (rad)	*ϕ*_3_(0) (rad)	*ϕ*_4_(0) (rad)	*ϕ*_5_(0) (rad)	*ϕ*_6_(0) (rad)	MTE (m)
1	1.7808	3.0571	2.8571	-2.4195	-1.1231	0.5936	1.8542E-05
2	1.7520	2.9731	2.9369	-2.6524	-1.2066	0.6614	2.0894E-05
3	1.4900	3.0006	2.9522	-2.3650	-0.9329	0.8299	2.1500E-05
4	1.6568	2.8675	2.9552	-2.5853	-0.9790	0.7477	2.1968E-05
5	1.4163	3.0275	2.9753	-2.4459	-1.2389	0.8020	2.7942E-05
6	1.4709	3.0593	2.9470	-2.5757	-1.3248	0.8050	2.9143E-05
7	1.4392	3.0250	3.0254	-2.4775	-1.0088	0.8503	3.0142E-05
8	1.7449	2.8619	2.9329	-2.5141	-0.9476	0.8860	3.3964E-05
9	1.5767	2.9744	2.8686	-2.4016	-1.1283	0.6500	3.6414E-05
10	1.6229	3.2258	3.1434	-2.4330	-1.1217	0.6740	**6.0982E-05**

Note: Maximum error is bolded.

**Table 6:** RMPC errors obtained by PNN solver depicted in Eq. (55) with random initial angle ***ϕ*** at *τ* = 0 s.

No.	*δ*_1_	*δ*_2_	*δ*_3_	*δ*_4_	*δ*_5_	*δ*_6_
1	2.3111E-07	-2.4818E-07	6.6954E-07	**1.3117E-06**	-5.2651E-07	-9.4811E-08
2	2.6647E-08	-7.3997E-08	6.5575E-08	2.5393E-07	-3.0109E-07	2.4176E-08
3	9.3697E-08	-9.1936E-08	3.8480E-07	6.2443E-07	-9.5763E-08	-1.6635E-07
4	1.3104E-08	-3.8405E-08	1.6888E-07	3.2259E-07	-1.2615E-07	-7.9563E-08
5	4.5718E-08	-6.5114E-08	7.8157E-08	2.4661E-07	-2.0940E-07	-1.5023E-08
6	5.0144E-08	-7.8441E-08	-7.1491E-09	1.1646E-07	-2.9019E-07	5.1426E-08
7	2.9771E-08	-4.4630E-08	1.3096E-07	2.7437E-07	-1.1754E-07	-6.6795E-08
8	1.4422E-08	-5.4617E-08	2.3825E-07	4.3418E-07	-1.4413E-07	-1.3619E-07
9	1.1562E-07	-1.3208E-07	3.9546E-07	7.7569E-07	-2.8471E-07	-9.2618E-08
10	8.5205E-08	-1.0294E-07	2.7853E-07	6.1562E-07	-2.7149E-07	-7.9074E-08

Note: Values of $\delta_{i}$ represent $\left|\phi_{i}(kT)-\phi_{i}(0)\right|$ which is described in objective depicted in Eq. (34). Maximum error is bolded.

### 6.3 Discussion

The purpose of this subsection is to offer a thorough examination and explanation of the outcomes, which is presented below.

• **Accuracy performance**: The accuracy of the model is evaluated in two respects. The first is the MTE ${\left\|r_{\text{d}}-r_{\text{a}}\right\|}_{2}\leq 10^{-4}$ m (illustrated in Table 5) and RMPC error $|\phi_{i}(kT)-\phi_{i}(0)|\leq 10^{-5}$ rad (illustrated in Table 6). It is apparent that the firstly presented solver achieves high-accuracy results as expected.
• **Validation data**: For verification purposes, ten sets of initial angles of joints are randomly selected, and the trajectory depicted in Eq. (56) is utilized.
• **Verification metrics**: The performance and effectiveness of the PNN solver depicted in Eq. (55) are evaluated using MTE and RMPC errors.
• **Evidential analysis**: The proof of convergence and accuracy for the PNN solver depicted in Eq. (55) is proven based on Theorems 1 and 2 in [69].
• **Discussion on limitations**: First, a theoretical kinematic model is assumed, disregarding certain complexities that may exist in real-world scenarios. Second, it is crucial to take into account the versatility and adaptability of the control strategy to effectively accomplish multiple tasks. These limitations offer opportunities for future exploration.
• **Impact assessment**: Implementing the firstly presented schemes in real RM systems holds the potential to improve their performance, efficiency, and adaptability. This can result in enhanced automation across diverse industries.
• **Cost-benefit analysis**: The cost of the PNN solver depicted in Eq. (55) for an RM encompasses both memory occupancy and CPU time occupancy. In the experiment using randomly selected initial values, the MATLAB memory usage shows that the total memory occupancy does not exceed 2,099,568 bytes. In terms of time occupancy, the program's execution time is quantified using MATLAB's Profile Viewer tool in the same experiment. The mean execution time is less than 5 s, which is substantially shorter than the usual execution time of 30 s for an RM to accomplish a trajectory-tracking task.
• **Fairness of decisions and different perspectives**: Firstly, to ensure fairness, the experiment in this research employs randomly selected initial values to test the model. Additionally, the path curves that the RM tracks are frequently encountered in the manufacturing of components. Thirdly, the convergence and stability of the PNN solver depicted in Eq. (55) are supported by rigorous mathematical proofs, offering robust interpretability and verifiability.
• **Mechanistic understanding and underlying phenomena**: A theoretical framework, i.e., ZEF-I2I, is introduced and implemented to address the motion planning optimization problem of RMs under multiple constraints. This framework consolidates various constraints and performance metrics at the velocity-tier and acceleration-tier. To validate the accuracy and reliability of the firstly presented solver, a comprehensive analysis is carried out by comparing and contrasting the theoretical data with the experimental data. Furthermore, additional experiments are performed to validate the firstly presented approach, utilizing ten randomly selected sets of initial values.
• **Interpreting evaluated results with respect to goals of the research**: The primary goals of this research are to enable the RM to track a trajectory, avoid obstacles, adhere to velocity and acceleration constraints, and meet the requirements of RMPC. In this research, all of these goals are successfully accomplished, and the corresponding results are illustrated in Figures and Tables in Subsections 6.1 and 6.2.
• **Limitations of evaluation**: The experimental results presented in this study are obtained through simulations, ensuring that they are not influenced by subjective biases or personal preferences of the researchers. Meanwhile, the selection of samples is entirely random, and the results are generated by the MATLAB program, making them fully reproducible and bias-free. Therefore, the observations obtained in the experiments precisely reflect the concept/phenomenon under investigation.
• **Alternative explanations**: Various conditions, such as environmental noises, mechanical loads on RMs, or specific conditions related to the tasks, may have influenced the outcomes. In this scenario, it is essential to establish constraint conditions based on physical laws and develop specific control methods to mitigate the impact of environmental noise [33], [38], [70].
• **Comparison with similar programs**: When contrasting the firstly presented schemes with conventional algorithms [68], [57], the experimental outputs clearly demonstrate the significant superiority of the firstly presented schemes.
• **Consistency with theories supported by previous research**: The literature review provided in Section 1, theoretical comparison presented in Sections 2 through 5, and results obtained in Subsections 6.1 and 6.2, broaden the context of the research findings and elucidate the connection between the research and the existing literature.
• **Inclusion of uncertainty in data and achieved outputs**: In real-world production lines, data uncertainty is influenced by disturbances in the machining trajectory. Following the methods outlined in [71], statistical approaches can be utilized for data processing, or filtering techniques can be utilized to mitigate the influence of noise. With respect to the uncertainty of output, deep learning models can be utilized to quantify uncertainties while considering their potential effects and limitations.

Based on the aforementioned analysis, one evidently summarizes that the conventional equivalency of bound constraints in the acceleration-tier is insufficiently effective. Meanwhile, the experimental simulation performed on an RM validates the accuracy of the theoretical model. This experimental validation serves as compelling evidence that supports the firstly presented approach and strengthens its practicality in real-world scenarios.

## 7 Conclusion

In this paper, the ZEF-E2E and ZEF-I2I have been introduced. Three typical subtypes of ZEF-E2E and ZEF-I2I have been presented, investigated, and proven. Meanwhile, the presented ZEF-E2E and ZEF-I2I have been applied for motion planning optimization of a Kinova Jaco2 RM. To achieve the four motion planning objectives, two schemes have been firstly presented to consolidate constraints from different tiers into a single tier. Thorough and comparative experiments have been carried out to extensively validate the accuracy of the ZEF-I2I and ZEF-E2E. Comparative experimental results have revealed that the method based on the ZEF-I2I has advantages over the conventional method with respect to usability, effectiveness, and robustness.

## Funding statement

This work is supported by multiple funding sources, including:

• The 10.13039/501100001809National Natural Science Foundation of China (No. 61976230).
• The Project Supported by Guangdong Province Universities and Colleges Pearl River Scholar Funded Scheme (No. 2018).
• The Key-Area Research and Development Program of Guangzhou (No. 202007030004).
• The Shenzhen Science and Technology Plan Project (No. JCYJ20170818154936083).
• The Research Fund Program of Guangdong Key Laboratory of Modern Control Technology (No. 2017B030314165).

## CRediT authorship contribution statement

**Dongqing Wu:** Conceptualization, Data curation, Formal analysis, Investigation, Methodology, Software, Visualization, Writing – original draft, Writing – review & editing. **Yunong Zhang:** Conceptualization, Funding acquisition, Investigation, Methodology, Project administration, Resources, Supervision, Validation, Writing – review & editing.

## Declaration of Competing Interest

The authors declare that they have no known competing financial interests or personal relationships that could have appeared to influence the work reported in this paper.

## Data Availability

No data was used for the research described in the article.
